# Mesenchymal stromal cells modulate survival and regeneration of human hematopoietic stem cells via PGE2/cAMP signaling

**DOI:** 10.1038/s41419-026-08502-w

**Published:** 2026-03-03

**Authors:** Siva Sai Naga Anurag Muddineni, Chen Katz-Even, Adi Zipin-Roitman, Diana Rasoulouniriana, Debanjan Singha Roy, Rabeaa Aborgies, Katia Beider, Yael Raz, Neta Solomon, Gal Hershkovitz, Yael Shulman, Rony Chen, Eviatar Weizman, Claudia Waskow, Arnon Nagler, Michael Milyavsky

**Affiliations:** 1 Department of Pathology, Tel Aviv University, Tel-Aviv, Israel; 2 Gray Faculty of Medical & Health Sciences, Tel Aviv University, Tel-Aviv, Israel; 3 Hematology Division, Chaim Sheba Medical Center, Tel-Hashomer, Israel; 4 Department of Obstetrics and Gynecology, Gynecologic Oncology Division, Lis Maternity Hospital, Tel Aviv Sourasky Medical Center, Tel-Aviv, Israel; 5 Helen Schneider Hospital for Women, Rabin Medical Center, Petach-Tikva, Israel; 6 G-INCPM, Weizmann Institute of Science, Rehovot, Israel; 7 Leibniz Institute on Aging, Fritz Lipmann Institute, Jena, Germany; 8 Institute of Biochemistry and Biophysics, Faculty of Biological Sciences, Friedrich-Schiller-University, Jena, Germany

**Keywords:** Haematopoietic stem cells, Mesenchymal stem cells, Stem-cell niche, Self-renewal

## Abstract

Ionizing radiation and chemotherapy significantly impair hematopoietic stem and progenitor cell (HSPC) function, increasing the risk of bone marrow failure and secondary malignancies. Mesenchymal stromal cells (MSCs), critical regulators within the hematopoietic niche, maintain HSPC quiescence, self-renewal, survival, and differentiation. However, the specific pro-regenerative signaling pathways activated by MSCs in human HSPCs remain incompletely defined. Here, we show that bone marrow-derived MSCs effectively suppress irradiation-induced apoptosis and preserve the in vivo repopulation capacity of human HSPCs. Transcriptomic analysis of HSPCs revealed a pronounced upregulation of CREB target genes following MSC co-culture, consistent with increased activation of the cAMP/CREB signaling pathway. Mechanistically, MSC-secreted prostaglandin E2 (PGE2) emerged as a key mediator of cAMP induced response in HSPCs. MSC-derived PGE2 preferentially inhibited IR-induced apoptosis in quiescent HSPCs, whereas pharmacological activation of cAMP pathway with Forskolin and IBMX (Forskolin/IBMX) robustly protected both quiescent and cycling HSPCs. Notably, the protective effect of Forskolin/IBMX persisted for up to 72 hours post-irradiation and significantly enhanced HSPC self-renewal. At the molecular level, we revealed reduced pro-apoptotic ASPP1 and PUMA expression, elevated p21 and stabilized anti-apoptotic MCL1 and BCL-XL proteins in human HSPCs treated with cAMP pathway agonists. Overall, our findings highlight the pivotal role of PGE2/cAMP/CREB signaling axis as a central mediator of MSC-mediated protection of human HSPCs under genotoxic stress and identify pharmacological cAMP activation as a promising strategy to protect human HSPCs against DNA damage-induced hematotoxicity.

## Introduction

Hematopoietic Stem and Progenitor Cells (HSPCs) are rare bone marrow (BM) cells positioned at the apex of the hematopoietic hierarchy. They possess the unique ability to self-renew and differentiate into multiple blood cell lineages. These regenerative properties are crucial for lifelong blood production, the success of autologous or allogeneic HSPC transplantation, and the regeneration of hematopoiesis following ionizing radiation (IR) or chemotherapy [1, 2].

The hematopoietic system is among the most radiosensitive tissues in our body. DNA damage to HSPCs critically limits hematopoietic regeneration by inducing apoptosis, impairing self-renewal, and causing aberrant differentiation. Patients with inherited defects in DNA damage response genes, as well as murine models of these diseases, frequently exhibit marked genomic instability, progressive BM failure due to impaired HSC function, and an increased incidence of hematopoietic malignancies [3]. A comprehensive understanding of the molecular pathways that regulate HSPC survival, maintenance, and regeneration under genotoxic stress is critical for developing therapeutic strategies to mitigate these adverse outcomes.

Prior work, including our own, have established that irradiated human HSPCs undergo rapid apoptosis and exhibit a pronounced loss of long-term repopulation capacity [4–7]. Genetic approaches have implicated key regulators such as p53, ASPP1, BCL-2, and MCL1 in the HSPC response to genotoxic injury [4, 8–10].

The heightened susceptibility of hematopoietic cells to genotoxic stress has driven pioneering studies in murine models to identify factors that promote HSPC regeneration. These studies implicated numerous BM niche-secreted factors in mitigating DNA damage-induced HSPC injury, including SCF [11, 12]. EGF [13, 14]. VEGF [15]. Angiopoietin [16]. Pleiotrophin [17–19]. Dickkopf-1 [20]. Angiogenin [21]. FGF [22, 23]. and PGE2 [24]. However, when tested on human HSPCs, key factors such as SCF, FLT3L, TPO, and EGF failed to inhibit DNA damage-induced apoptosis in vitro or protect human hematopoiesis in immunodeficient xenograft models [25]. These discrepancies underscore species-specific differences in irradiation susceptibility [26]. which can stem from variations in transcriptional responses [27]. cytokine requirements, telomere/telomerase biology, and the inbred nature of murine strains, complicating the direct extrapolation of rodent findings to humans [28–30].

The BM microenvironment consists of a variety of non-hematopoietic stromal cells that engage in dynamic crosstalk with HSPCs within specialized niche structures [31, 32]. Mesenchymal stromal cells (MSCs), a critical niche component, support HSPC long-term regenerative capacity in both in vivo and ex vivo settings [33–36]. Substantial research has explored MSCs therapeutic potential in protecting the hematopoietic system from IR-induced injury [35–43]. While these studies have demonstrated increased survival of irradiated hematopoietic cells in co-culture, the identity of MSC-derived pro-survival factors, the molecular pathways engaged in human HSPCs, and their functional impact in vivo remains largely unknown.

Prostaglandin E2 (PGE2) is a bioactive lipid that regulates vertebrate HSPCs via EP2 (PTGER2) and EP4 (PTGER4) Gs-protein-coupled receptors, activating the cyclic AMP (cAMP) signaling pathway [44–46]. The cAMP-responsive element-binding protein (CREB), a master transcription factor activated by elevated cAMP levels, mediates PGE2-dependent transcriptional responses [47]. Short-term (2 h) PGE2 exposure enhanced HSPC migration, survival under cytokine deprivation, homing ability, and human chimerism in xenograft models [48–50]. but failed to improve neutrophil recovery in a clinical trial [49]. Likewise, prolonged (24–48 h) ex vivo PGE2 stimulation before xenotransplantation did not enhance human chimerism [51–53]. While, systemic PGE2 administration pre-IR rescued murine HSPC function [54]. It showed no protective effect in humanized mice [25]. Thus, whether physiological PGE2 mediates MSC-mediated pro-survival signaling and promotes human HSPC regeneration after DNA damage remains unresolved.

Here we demonstrate that MSC-secreted PGE2 activates cAMP signaling via EP2 and EP4 receptors in HSPCs, preventing IR-induced apoptosis by rapidly modulating ASPP1, p21, and PUMA expression. Notably, pharmacological cAMP activation with small-molecule agonists replicated MSC-mediated protection, and markedly enhanced HSPC functionality ex vivo and in vivo.

## Materials and methods

Experimental procedures are described in detail in Supplemental Materials.

## Results

### MSCs promote stemness and anti-inflammatory transcriptional program in human HSPCs

**Fig. 1:**
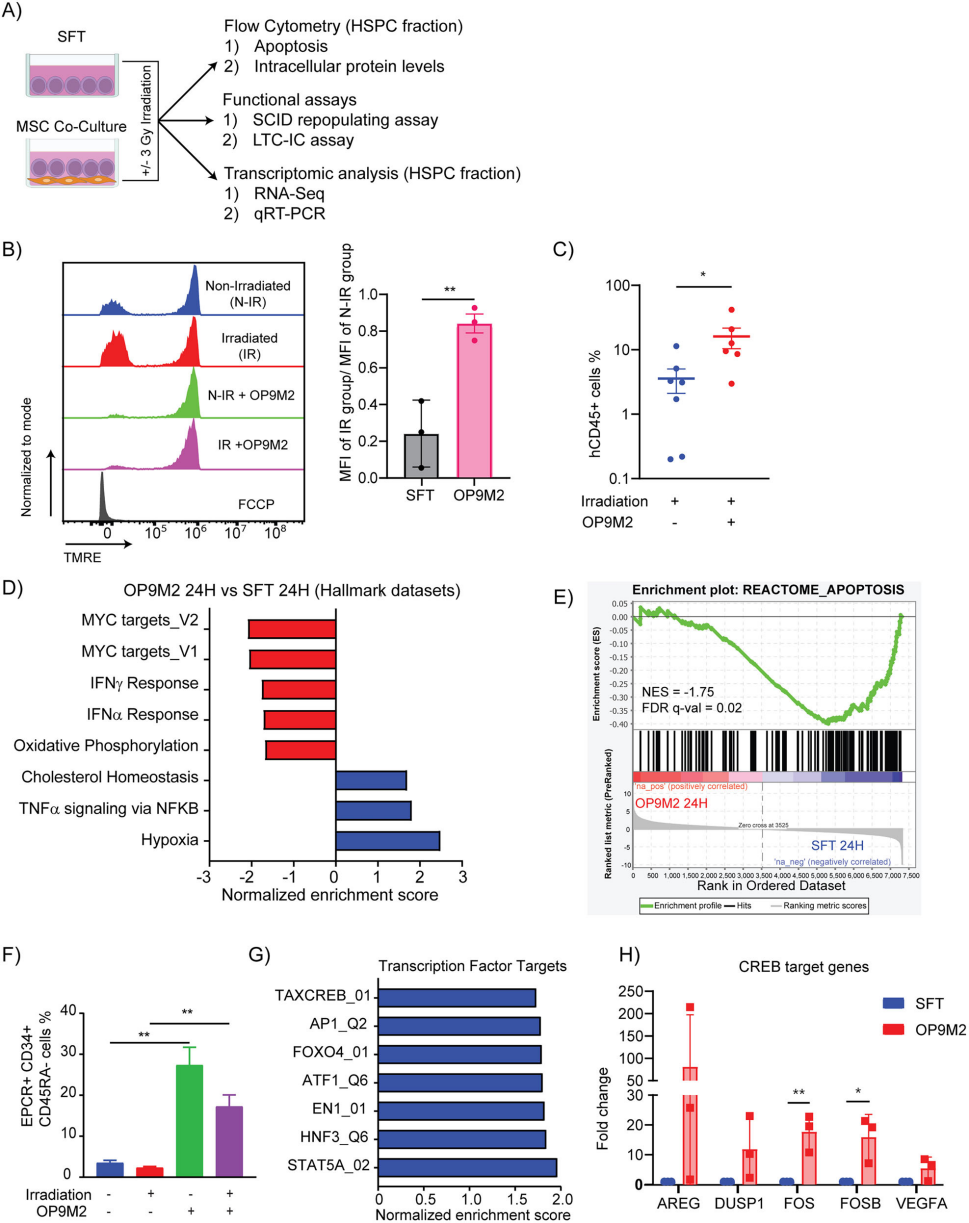
MSCs activate a CREB-associated stemness and anti-apoptotic transcriptional program to protect irradiated human HSPCs. **A** Schematic of the experimental design to evaluate HSPC survival and functionality following co-culture with MSCs post-irradiation. **B** Analysis of mitochondrial membrane potential in HSPCs under the indicated conditions. Left: representative TMRE histograms; right: quantification expressed as the ratio of mean fluorescence intensity (MFI) between irradiated and non-irradiated cells (*n* = 3). **C** In vivo assessment of HSPC functionality. CB CD34+ HSPCs cultured for 24 h under the indicated conditions were transplanted retro-orbitally into NSGW41 mice. Percentage of human CD45+ cells in murine bone marrow 15 weeks post-transplant. Each symbol represents an individual recipient; horizontal bars denote the mean (*n* = 2). **D** GSEA showing significant enrichment of hallmark gene sets in HSPCs co-cultured with OP9M2 MSCs compared to cytokine-only culture. **E** Gene Set Enrichment Analysis (GSEA) plot illustrating the enrichment of apoptosis pathway genes in HSPCs co-cultured with OP9M2 MSCs versus those cultured in serum-free medium with cytokines. **F** Flow cytometric quantification of EPCR expression on CD34 + CD45RA- HSPCs cultured under the indicated conditions for up to 96 h (*n* = 3). **G** GSEA demonstrating significantly enriched transcription factor target gene sets in HSPCs co-cultured with OP9M2 MSCs relative to cytokine-only conditions. **H** qRT-PCR analysis validating upregulation of CREB target genes in HSPCs co-cultured with OP9M2 MSCs compared to controls (*n* = 3). Data are mean ± SEM. Statistical significance was assessed by unpaired two-tailed Student’s *t*-test.

To decipher the molecular mechanisms underlying MSC-mediated protection against genotoxic stress, we established a niche mimicking human CD34^+^ -MSC co-culture system (Fig. 1A). As expected, upon IR the more immature fraction defined here as CD34 + CD38-CD45RA- HSPCs showed a significant increase in Annexin^+^ apoptotic cells, whereas co-culture with human BM-MSCs completely prevented apoptosis (Supplemental Fig. 1B). Due to the limited availability of primary human BM-MSCs, we used the well-characterized murine OP9M2 MSC cell line to support and extend results obtained with human MSCs [55, 56]. (Supplemental Fig. 1A). Co-culturing HSPCs with OP9M2 cells prevented apoptosis induced by IR or Etoposide treatments (Supplemental Fig. 1B, D).

Given the key role of mitochondria in cell functionality, we assessed mitochondrial mass and integrity in HSPCs cultured with cytokines or in contact with OP9M2. In the frame of 24 h, we detected no changes in mitochondrial mass or mitochondrial membrane potential between HSPCs cultured alone or in presence of MSCs. In contrast, while IR did not affect mitochondrial mass, it induced mitochondrial depolarization in HSPCs cultured alone, correlating with increased apoptosis. Co-irradiated MSCs preserved mitochondrial polarization in HSPCs, consistent with reduced rate of apoptosis (Fig. 1B; Supplemental Fig. 1E, 1F). We observed no mitochondrial transfer from MSCs to HSPCs, ruling out this possibility from MSC mediated protection (Supplemental Fig. 2A–C).

To functionally evaluate the impact of MSC-mediated protection on HSPC regenerative potential, we performed in vitro and in vivo assays. In long-term culture-initiating cell (LTC-IC) assays, irradiated HSPCs in monoculture exhibited significantly reduced colony-forming potential. In contrast, MSC co-cultures enhanced LTC-IC frequency of irradiated CD34^+^ cells by nearly five-fold, indicating maintained clonogenicity (Supplemental Fig. 3A). We next evaluated the in vivo engraftment ability of irradiated HSPCs using immunodeficient mouse models. HSPCs cultured without MSCs post-IR engrafted poorly (~3% human CD45+ cells), whereas MSC co-culture substantially improved engraftment to ~16% (Fig. 1C). Notably, multilineage differentiation capacity and splenic homing of HSPCs remained unaltered, suggesting that MSC interaction preserved the fundamental properties of hematopoietic stem cells, including lineage potential and trafficking competency (Supplemental Fig. 3B, D).

To elucidate transcriptional programs underlying MSC-mediated protection, we performed RNA-seq on CD34 + CD38-CD45RA- HSPCs cultured alone or in direct contact with MSCs for 24 h. MSC co-culture led to a robust transcriptional shift characterized by significant upregulation of AP-1 transcription factors (FOS, FOSB, FOSL2, JUN) and metabolic genes involved in glycolysis (ALDOC, PFKFB4). Concurrently, transcripts encoding pro-inflammatory cytokines and chemokines, including IL1B, STAT1, CCL1, and CCL2, were downregulated (Supplemental Fig. 4A). Gene set enrichment analysis (GSEA) revealed suppression of stress-associated signatures, including apoptosis, unfolded protein response, and oxidative phosphorylation, alongside repression of MYC target genes and both type I and II interferon response pathways (Fig. 1D, E). In parallel, MSC-exposed HSPCs showed enrichment of stemness-associated programs, including HSC quiescence, glycolytic metabolism, and transcriptional signatures of regenerative EPCR+ HSPCs (Supplemental Fig. 4C). Flow cytometry confirmed a marked increase in EPCR surface expression in MSC co-cultured HSPCs, consistent with expansion of functionally superior regenerative subsets (Fig. 1F).

### MSCs activate cAMP/ CREB signaling pathway to protect human HSPCs

**Fig. 2:**
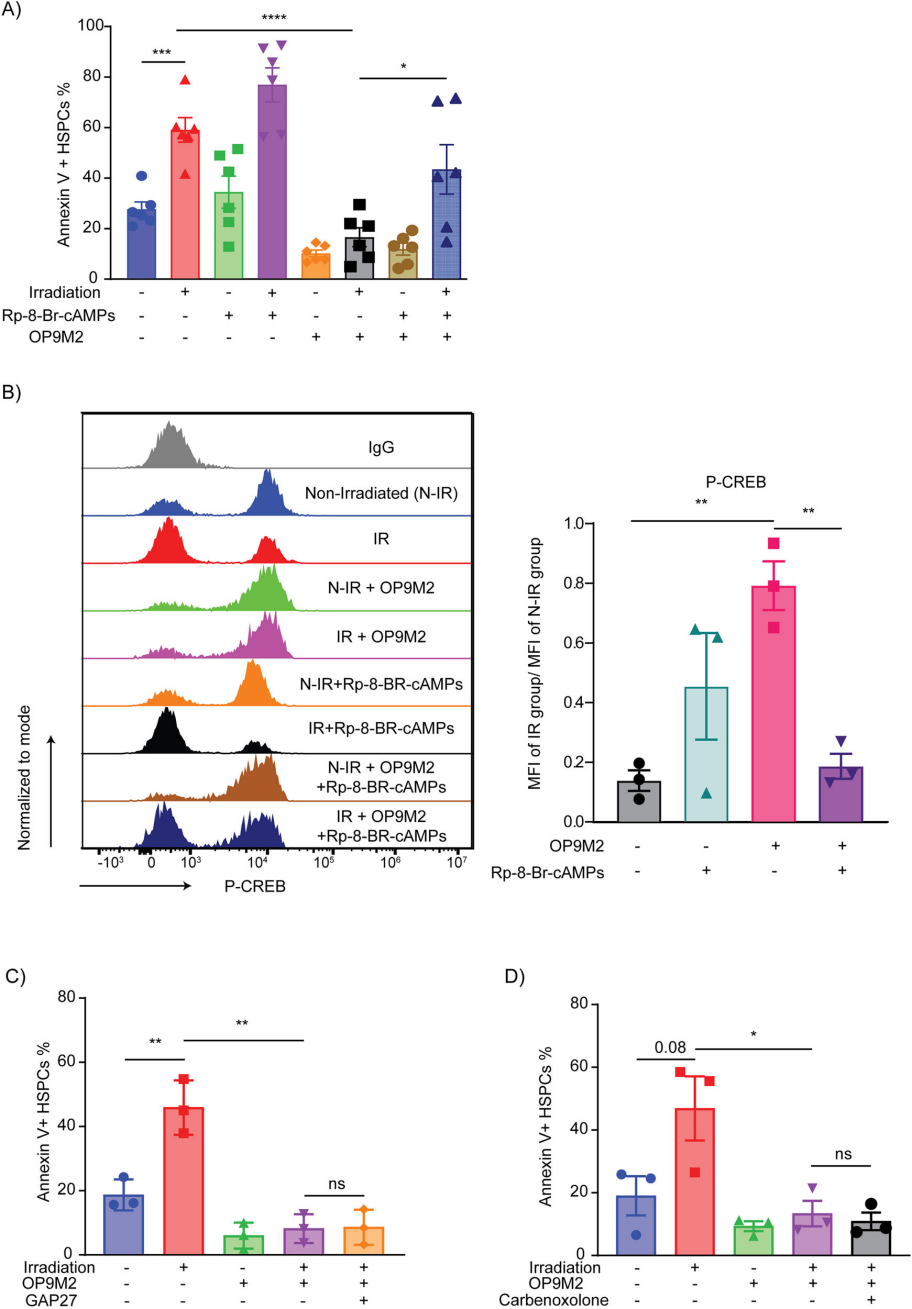
MSCs activate the cAMP/CREB signaling pathway to protect human HSPCs. **A** CD34+ cells in mono- or MSC co-culture were pre-incubated with Rp-8-Br-cAMPS followed by IR (3 Gy). Fraction of Annexin V + CD34 + CD38-CD45RA- HSPCs was determined 24 h after IR (*n* = 6). **B** Flow cytometric analysis of phospho-CREB (Ser133) levels in CB CD34 + CD38-CD45RA- HSPCs after 24 h under the indicated conditions (*n* = 3). **C, D** CB CD34+ cells cultured for 24 h in the presence of gap junction inhibitors (Carbenoxolone and GAP27), fraction of Annexin V + CD34 + CD38-CD45RA- HSPCs was determined 24 h after IR (*n* = 3). Data are mean ± SEM. Statistical significance was assessed by unpaired two-tailed Student’s *t*-test.

To identify upstream regulators responsible for the observed transcriptional shifts, we performed GSEA using the Transcription Factor Targets (TFT) gene set collection. We found strong predicted activation of bZIP family transcription factors, notably CREB1 and ATF1, along with the AP-1 complex (Fig. 1G; Supplemental Fig. 4B, 4D). Consistent with this, targeted HOMER analysis of promoter regions showed modest enrichment of the canonical CRE motif (TGACGTCA) and a CRE-like/AP-1 motif (TGACTCA) in upregulated genes relative to background (Table 1). CREB1 is a well-established downstream effector of cAMP signaling, known to regulate stress response genes and survival in a cell type specific manner. We validated upregulation of canonical CREB target genes in both OP9M2 and hBM-MSC co-cultured HSPCs via qRT-PCR, consistent with transcriptome-level predictions (Fig. 1H; Supplemental Fig. 4E). Importantly, treatment with the PKA inhibitor Rp-8-Br-cAMPs blocked MSC-mediated protection and reduced CREB phosphorylation in HSPCs, indicating that MSC-derived cues induce CREB activation through a cAMP-dependent mechanism (Fig. 2A, B; Supplemental Fig. 5A–5C).

**Table 1:** HOMER motif enrichment for the canonical CRE motif (TGACGTCA) and the CRE-like/AP-1 motif (TGACTCA) in promoters of genes upregulated in HSPCs upon OP9M2 MSC co-culture.

Motifs	P value	Target (%)	Background (%)
**AP1**	**1e-3**	**12.05**	**5.29**
**CREB1**	**1e-2**	**4.22**	**1.13**

To test whether cAMP is transferred from MSCs to HSPCs through direct cell-cell contact, we disrupted gap junctions using Carbenoxolone and GAP27 inhibitors. Neither agent affected MSC-mediated protection, suggesting that intracellular cAMP is not shuttled through connexin-based channels (Fig. 2C, D; Supplemental Figs. 6A, B).

### PGE2 secreted by BM-MSCs promote HSPCs survival via EP2 and EP4 receptors

**Fig. 3:**
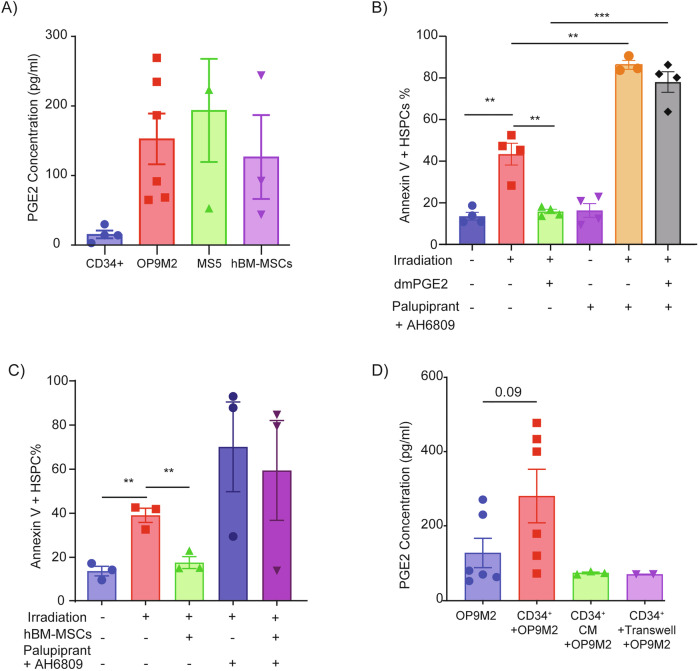
BM-MSCs secrete PGE2 to protect human HSPCs from IR-induced apoptosis. **A** ELISA-based quantification of PGE2 in conditioned media collected 24 h after growing different cell types in SFEM SFT medium (*n* = 4). **B, C** Annexin V analysis of apoptosis in CB CD34 + CD38-CD45RA- HSPCs cultured for 24 h under the indicated conditions, with or without dmPGE2/ hBM-MSCs and selective EP receptor antagonists (AH6809 for EP1–3 and Palupiprant for EP4) (*n* = 4). **D** Comparison of PGE2 secretion by OP9M2 MSCs when co-cultured with CD34+ cells either in direct contact or separated by a 0.4μm transwell (*n* = 3).

We next aimed to identify factors secreted by MSCs that trigger can cAMP/CREB activation in HSPCs. Based on literature mining and receptor expression analysis, we compiled a list of Gs-coupled ligands known to be secreted by MSCs and examined their cognate receptor expression on human HSPCs. Among all candidates, PGE2 emerged as the most likely mediator. EP2 and EP4, two Gs-coupled PGE2 receptors, were robustly expressed on HSPCs, while other Gs-coupled receptors exhibited negligible expression (Table 2). PGE2 levels measured in culture supernatants confirmed that PGE2 was abundantly secreted by different MSCs but not by CD34+ HSPCs themselves (Fig. 3A). Importantly, pharmacological blockade of EP receptors using AH6809 (EP1-3 antagonist) or Palupiprant (EP4-specific antagonist) abrogated the cytoprotective effects of both MSCs and exogenously added dmPGE2 (Fig. 3B, C; Supplemental Fig. 7A, 8A-C). AH6809 and Palupiprant treatment did not alter PGE2 secretion by MSCs (Supplemental Fig. 8D), further supporting a direct signaling mechanism.

**Table 2:** Potential cAMP activating ligands and their receptors in the context of MSC-HSPC crosstalk.

BM-MSC-secreted molecules reported to activate Gs coupled GPCRs	Cognate Gs coupled receptors	Receptor expression on CD34 + CD38-CD45RA- HSPCs
Prostaglandin E2 (PGE₂) [74].	PTGER2	Low
	PTGER4	Yes
Adenosine [75].	ADORA2A	Not expressed
	ADORA2B	Low
Parathyroid hormone-related protein (PTHrP) [76].	PTH1R	Not expressed

Only ligands capable of stimulating Gs coupled receptors and produced by MSCs were considered.

Interestingly, co-culture with HSPCs enhanced PGE2 secretion from OP9M2 MSCs, revealing a positive feedback loop and highlighting the reciprocal nature of MSC-HSPC communication (Fig. 3D). Thus, PGE2 emerges as the dominant MSC-secreted factor that signals through EP2 and EP4 to activate cAMP/CREB and safeguard human HSPCs.

### Pharmacological activation of cAMP/CREB signaling pathway protects human HSPCs from IR induced apoptosis

**Fig. 4:**
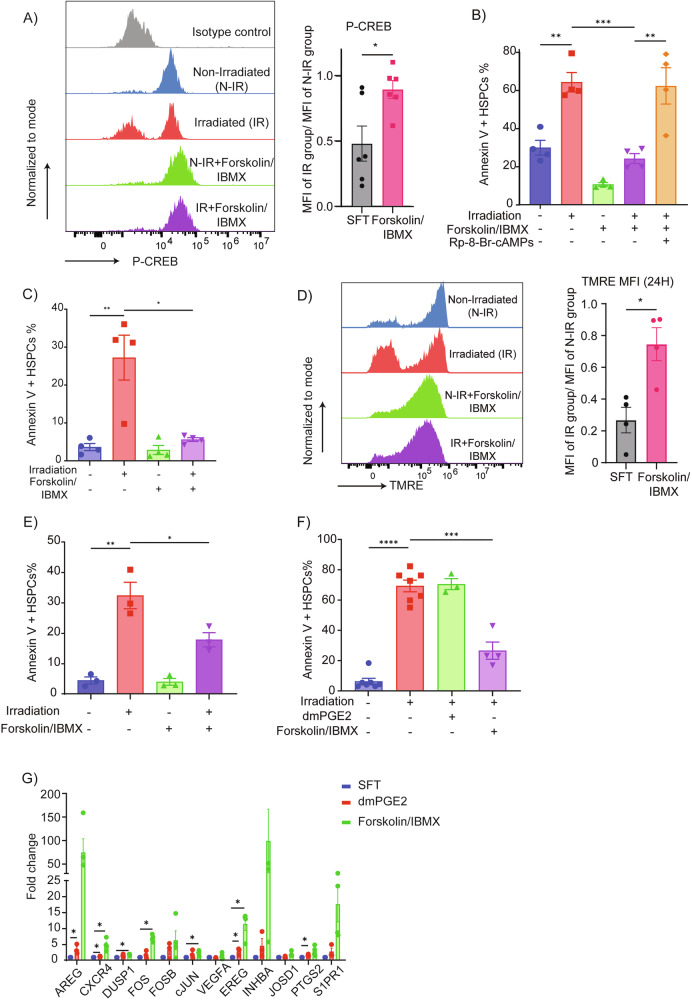
Pharmacological activation of the cAMP/CREB signaling pathway protects human HSPCs from IR-induced apoptosis. **A** Flow cytometric quantification of phospho-CREB (Ser133) levels in CB CD34 + CD38-CD45RA- HSPCs after 24 h of treatment with Forskolin/IBMX, demonstrating sustained CREB activation post-irradiation (*n* = 4). **B** Annexin V staining showing that Forskolin/IBMX pre-treatment significantly reduces IR-induced apoptosis in CB CD34 + CD38-CD45RA- HSPCs; this protective effect is abrogated by the cAMP antagonist Rp-8-Br-cAMPs (*n* = 4). **C** Apoptosis analysis in human BM-derived CD34 + CD38-CD45RA- HSPCs cultured under the indicated conditions for 24 h (*n* = 3). **D** TMRE staining demonstrating preserved mitochondrial membrane potential in Forskolin/IBMX-treated CD34 + CD38-CD45RA- HSPCs: representative histograms (left) and MFI quantification (right) (*n* = 3). **E** Annexin V analysis of apoptosis in cycling CB HSPCs (pre-cultured for 72 h) after 24 h exposure to the indicated treatments (*n* = 3). **F** Quantification of apoptosis in CB CD34+ cells cultured for 72 h post-irradiation under the indicated conditions (*n* = 3). **G** qRT-PCR analysis of CREB target gene expression in CD34+ cells treated with either dmPGE2 or Forskolin/IBMX for 20 h (*n* = 4). Data are mean ± SEM. Statistical significance was assessed by unpaired two-tailed Student’s *t*-test.

To directly activate cAMP signaling and assess its cytoprotective potential, we treated HSPCs with Forskolin (an adenylyl cyclase activator) and IBMX (a broad phosphodiesterase inhibitor). This combination synergistically restored P-CREB levels in irradiated HSPCs (Fig. 4A; Supplemental Fig. 10A). Importantly, Forskolin/IBMX significantly reduced IR-induced apoptosis in neonatal and adult BM HSPCs, an effect that was reversed by PKA inhibition (Fig. 4B, C; Supplemental Fig. 9B, 9D). Forskolin/IBMX treatment of HSPCs also preserved mitochondrial membrane potential following IR (Fig. 4D; Supplemental Fig. 10D).

Interestingly, we observed that both OP9M2 and dmPGE2 were unable to rescue cycling HSPCs from IR-induced apoptosis (Supplemental Fig. 10E). Unlike dmPGE2, Forskolin/IBMX conferred protection to both quiescent and actively cycling HSPCs over 72 hours, indicating a broader therapeutic window and sustained efficacy (Fig. 4E, F). Furthermore, induction of CREB-responsive genes was more sustained with Forskolin/IBMX than with dmPGE2. (Fig. 4G; Supplemental Fig. 10F).

### Pharmacological activation of the cAMP/CREB pathway enhances the function of irradiated human HSPCs

**Fig. 5:**
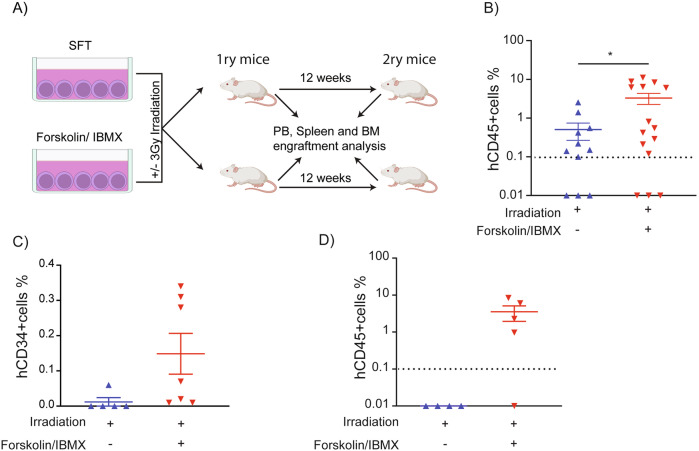
Pharmacological activation of the cAMP/CREB signaling pathway enhances engraftment, self-renewal, and protects engrafted human hematopoietic cells from IR-induced apoptosis in vivo. **A** Schematic representation of the experimental design to assess HSPC functionality following irradiation and Forskolin/IBMX treatment ex vivo. **B** Human chimerism in the bone marrow of NSGW41 mice 12 weeks post-injection of control or Forskolin/IBMX-treated and irradiated (3 Gy) hCD34+ cells (each symbol represents an individual recipient; horizontal bars denote the mean; *n* = 2). **C** Quantification of immature (hCD34 + ) cell frequency in the bone marrow of mice transplanted with control versus Forskolin/IBMX-treated CD34⁺ cells (*n* = 2). **D** Human chimerism in the bone marrow of secondary recipient mice following transplantation of total bone marrow from primary recipients (each symbol represents an individual recipient; horizontal bars denote the mean). Data are mean ± SEM. *P* values determined by one-way ANOVA with Tukey’s multiple comparisons test.

To specifically evaluate the intrinsic effects of cAMP-elevating drugs on the function of irradiated human HSPCs - independent of systemic influences that may arise in vivo - we pre-treated CD34+ cells with Forskolin/IBMX ex vivo, irradiated them and transplanted them into NSGW41 mice (Fig. 5A). Compared to vehicle-treated controls, Forskolin/IBMX-treated cells showed significantly higher levels of human chimerism in the bone marrow (6.6-fold increase) and successful engraftment in a higher proportion of recipients (12/15 vs. 4/11) (Fig. 5B, C; Supplemental Fig. 11B, C). In contrast, dmPGE2-treated HSPCs showed no significant benefit in engraftment capacity (Supplemental Fig. 11A). In secondary transplants, only Forskolin/IBMX-pretreated HSPCs retained multilineage reconstitution potential, confirming preserved stemness and self-renewal (Fig. 5D; Supplemental Fig. 11D).

To test whether systemic pharmacological activation of cAMP signaling could protect human hematopoietic cells, we injected human CD34+ cells into NSG mice. Twelve weeks post-engraftment, mice were stratified into three groups based on peripheral blood chimerism and treated with either 1) DMSO (non-irradiated), 2) DMSO followed by total body irradiation (TBI, 3 Gy) or 3) Forskolin/IBMX followed by TBI (3 Gy), one hour later (Supplemental Fig. 12A). Twenty-four hours post IR, flow cytometric analysis of bone marrow revealed no significant difference in total human CD45+ chimerism across groups, but TBI markedly increased apoptosis in human CD45+ hematopoietic cells (Supplemental Fig. 12B, C). Notably, Forskolin/IBMX pre-treatment significantly reduced this irradiation-induced apoptosis compared to vehicle-treated controls, indicating that transient in vivo activation of the cAMP pathway can attenuate acute genotoxic injury to human hematopoietic cells within the bone marrow niche (Supplemental Fig. 12C).

TBI is known to induce pro-inflammatory cytokines that can also trigger inflammatory cell death pathways in bone marrow cells in addition to apoptosis [57]. Thus, we next examined the inflammatory milieu after irradiation. TBI caused only a modest, rise in human IFNγ and no change in TNFα (Supplemental Fig. 12D, E), and Forskolin/IBMX did not further elevate IFNγ, indicating that systemic cAMP activation does not alter IR-induced cytokine release under radiation regiment employed. Despite reducing Annexin+ cell fraction after IR, pharmacological cAMP activation failed to preserve human long-term BM and splenic repopulating capacity in secondary recipients (Supplemental Fig. 12F, G), highlighting the complexity of the in vivo pathways ultimately regulating HSPC survival. Together, these long-term in vivo and in vitro data establish that pharmacological activation of cAMP signaling pathway can mimic pro-survival phenomenon of MSC co-culture and offers significant radioprotection on ex vivo irradiated human HSPCs.

### Pro-survival effects of cAMP agonists are dependent on functional anti-apoptotic MCL1/BCL-XL proteins and altered P53/ASPP1 program

**Fig. 6:**
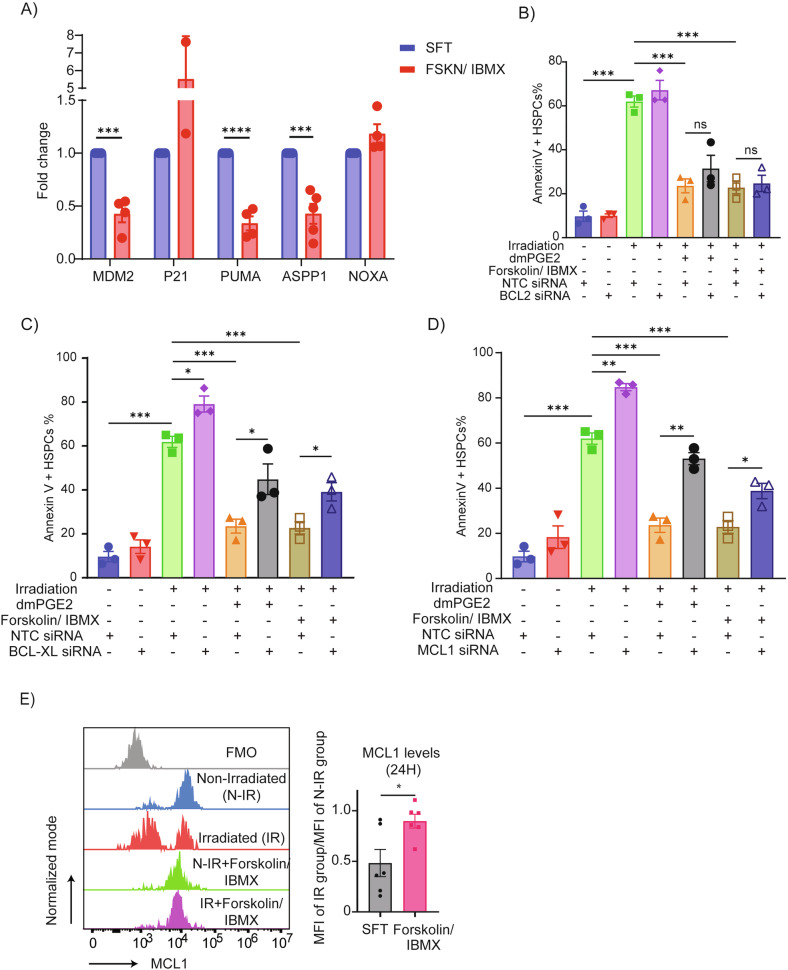
Pro-survival effects of cAMP/CREB signaling depend on functional MCL1 and BCL-XL proteins. **A** qRT-PCR analysis of selected p53 pathway-related genes in CD34+ cells treated with Forskolin/IBMX for 2 h (*n* = 4). CB CD34+ cells were nucleofected with non-targeting control (NTC) or siRNA pools targeting BCL2 (**B**), BCL-XL (**C**), or MCL1 (**D**) and allowed to recover for 24 h. Nucleofected cells were then exposed to the indicated treatments followed by apoptosis analysis in CD34 + CD38-CD45RA- HSPCs after 24 h (*n* = 3). **E** Flow cytometric analysis of MCL1 protein levels in CD34 + CD38-CD45RA- HSPCs cultured with or without Forskolin/IBMX for 24 h. Left: representative histograms; right: quantitative MCL1 expression (*n* = 6).

To identify downstream effector proteins mediating cAMP-induced radioprotection, we analyzed transcript and protein levels of pro- and anti-apoptotic BCL-2 family members. At early time points (3 hours post-treatment), MCL1 mRNA was strongly induced by both Forskolin/IBMX and dmPGE2, while BCL2 and BCL-XL transcripts were transiently suppressed (Supplemental Figs. 13A, C). By 20 hours, mRNA expression of MCL1 and BCL-XL returned to baseline, while BCL2 remained low (Supplemental Figs. 13D–F). In addition, we revealed that cAMP agonist treatment attenuated expression of selected p53 targets (e.g. MDM2, PUMA), and co-factors (e.g. ASPP1), while inducing cell cycle regulator p21 (Fig. 6A), collectively supporting an anti-apoptotic shift in transcriptional programming.

To test functional relevance, we used siRNA to knock down BCL2, BCL-XL, or MCL1 in HSPCs (Supplemental Figs. 13G–I). While BCL2 depletion had no significant effect, knockdown of either MCL1 or BCL-XL abolished the protective effects of Forskolin/IBMX and dmPGE2 (Fig. 6B–D), indicating their non-redundant roles. Intracellular flow cytometry analysis confirmed that Forskolin/IBMX and MSC co-culture stabilize MCL1 protein levels in irradiated HSPCs, whereas BCL2 and BCL-XL proteins remained unchanged (Fig. 6E; Supplemental Figs. 14A–C, 15A–C).

Finally, we assessed expression of Survivin (BIRC5), a known regulator of apoptosis previously implicated in PGE2 signaling [58]. Survivin transcript and protein levels were not significantly altered by dmPGE2 or Forskolin/IBMX treatment under steady-state conditions. However, IR-induced downregulation was reversed in OP9M2 MSC coculture and dmPGE2 conditions, suggesting a context-dependent stabilization (Supplemental Figs. 16A–D).

In summary, our integrative analysis demonstrates that dmPGE2 and Forskolin/IBMX conferred radioprotection depends on the stabilization of MCL1 and BCL-XL proteins in human HSPCs. This dual requirement underscores the complexity of IR induced apoptosis regulation in HSPCs and highlights the importance of a coordinated anti apoptotic signaling by cAMP elevation.

## Discussion

Our results establish that activation of cAMP signaling pathway potently inhibits DNA damage-induced apoptosis and enhances the long-term regenerative potential of human HSPCs. In an ex vivo niche-mimicking model, MSCs secrete PGE2 upon contact with HSPCs, engaging EP2 and EP4 receptors to suppress IR-induced apoptosis. Pharmacological modeling revealed that both PGE2 and the Forskolin/IBMX (Forskolin/IBMX) combination mitigate early apoptosis, but only Forskolin/IBMX enhanced the repopulation capacity of irradiated HSPCs in both primary and secondary transplantation assays. Mechanistically, cAMP agonists downregulated pro-apoptotic ASPP1 and PUMA, and upregulated MCL1 and p21, aligning with reduced apoptosis and enhanced survival. These findings demonstrate that physiological or pharmacological activation of cAMP signaling ameliorates IR-induced functional loss of human HSPCs.

Dissection of the MSCs impact on the transcriptome of irradiated human HSPCs is unique to this study and extends prior knowledge obtained from murine HSPCs isolated after whole body IR [9, 54]. Our transcriptomic analyses revealed that MSCs induces a distinct gene expression program in HSPCs, characterized by upregulation of stemness-associated transcription factors (e.g., FOS, JUN, EGR1 and CREB) and a concurrent suppression of pro-inflammatory, stress-related and proliferative pathways (e.g. IFNa, IFNg and MYC). Abrupt transcriptional induction of multiple stress pathways in human HSPCs ex vivo was recently proposed to negatively impact expansion and functionality [56, 59]. On the other hand, our transcriptomic analysis reveals that MSCs can stabilize human HSPCs ex vivo in the state reminiscent of their freshly isolated counterparts. Interestingly, MSC-induced transcriptional signature in HSPCs was not significantly affected by IR. Our transcriptomic results underscore niche signaling complexity and argue that MSCs can prime HSPCs for enhanced resilience to IR and other genotoxic insults.

While dissecting the possible mechanisms by which MSCs can elevate cAMP levels in human HSPCs, we excluded the involvement of mitochondrial transfer or gap junction mediated exchange [60, 61]. Instead, we found that both human and murine MSCs in contact with MSCs increased secretion of physiological cAMP agonist PGE2, which blocked IR-induced apoptosis and promoted regeneration. The hematopoietic supportive activity of MSCs is primarily attributed to the secretion of proteins such as CXCL12, SCF, ANGPT1 and Osteopontin amongst others. Our findings indicate that PGE2, a bioactive lipid as the predominant signaling mediator in the MSC secretome that protects HSPCs from IR induced apoptosis. Indeed, blocking the PGE2 receptors on HSPCs underscores the importance of BM-MSC derived PGE2 in regulating HSPC survival. Our data expands on the previous observations that PGE2 and its synthetic analogue dmPGE2 regulate HSPC survival [44, 58]. A deeper understanding of the mechanisms regulating PGE2 production and receptor expression on HSPCs will be essential for advancing MSC- or PGE2- based strategies to enhance HSPCs recovery.

Although the detailed mechanisms that account for the cell type specific control of DNA damage induced apoptosis by cAMP are still elusive, our results reveal that cAMP agonists can repress ASPP1 - the endogenous stimulator of p53-dependent apoptosis in cells, including HSPCs [4, 9]. In agreement with the established role of ASPP1 in modulation of p53 transcriptional response we observed coordinated decline in the expression of pro-apoptotic (PUMA) and upregulation of pro-survival genes (e.g. MCL1 and p21) upon HSPC treatment with cAMP agonists. This shift in p53 transcriptional response also provides plausible molecular mechanism for the observed block in IR-induced apoptosis (via decreased PUMA) and early cycling (via elevated p21) of mouse HSPCs upon injection of PGE2 [54]. Although cAMP agonists resulted only in partial blocking of PUMA induction, murine models posits that even 50% reduction in PUMA expression suffices to significantly protect from the lethal IR-hematotoxicity [6, 62]. Elevated activity of CRE-binding factors (e.g., CREB, ATF1 and AP1 complex) in HSPCs from MSCs co-culture or after treatment with cAMP agonists (this study) or of SIRT1 [63]. can additionally contribute to the diminished IR-induced apoptosis in HSPCs. Indeed, efficient transactivation of p53, as well as of CREB and NF-KB target genes, are dependent on their binding to p300 and CBP histone acetyltransferases [64]. Given limited cellular amounts of p300/CBP, higher levels of active CREB, as indicated by Ser133 phosphorylation in HSPCs, might destabilize pro-apoptosis gene regulatory programs [65]. and tilt HSPCs cell fate towards survival.

Our mechanistic studies further underscore the differential importance of anti-apoptotic BCL-2 family proteins in this process. Although early transcriptional responses to cAMP elevated treatments included a transient downregulation of BCL2 and BCL-XL, functional assays demonstrated that the radioprotective benefits of both PGE2 and Forskolin/IBMX depend critically on the stabilization of MCL1 and the activity of BCL-XL. Notably, while BCL-XL protein levels did not decrease immediately following irradiation, it is likely that BCL-XL undergoes rapid deamidation as an immediate response to IR stress [66, 67]. —a modification that may impair its function—and that its protein levels may decline at later time points. siRNA-mediated knockdowns confirmed that disruption of either MCL1 or BCL-XL, but not BCL-2, abrogated the protective effects of PGE2 and Forskolin/IBMX, emphasizing the need for a coordinated anti-apoptotic response in mitigating IR-induced damage. In addition to mediating cAMP pro-survival effects as uncovered here, elevated dependency of HSPCs on both BCL-XL and MCL-1 for their survival after DNA damage, as uncovered here, and by Erlacher group [8, 68]. represents a human specific phenomenon, as mouse counterparts dependent only on MCL-1 [69]. and would predict severe hematotoxicity upon clinical targeting with selective inhibitors.

Importantly, despite efficient IR-induced apoptosis abrogation in HSPCs by cAMP agonists ex vivo, total human engraftment (hCD45+ cells) remained markedly reduced, indicating in vivo reactivation of DNA damage induced mechanisms that limit HSPC proliferation. Remarkably, our re-transplantation experiments demonstrated that even few Forskolin/IBMX pre-treated irradiated HSPCs preserved significant self-renewal ability that was lost completely in the cytokine only cultured irradiated HSPCs. Moreover, they chart a critical time window in which modulation of DNA damage induced apoptosis can play a critical role for preservation of human HSPC functionality. These findings also independently support a concept obtained in transgenic animals with switchable p53 [70]. or deleted PUMA [71, 72]. suggesting that while transient inactivation of p53-dependent apoptosis can rescue myelosuppression, its delayed functional restoration suffices to suppress IR-induced and compensatory proliferation driven carcinogenesis.

While agents like dmPGE2 have shown promise in murine models [44, 54, 63]. our data indicates that direct modulation of the cAMP/CREB axis—via compounds such as Forskolin/IBMX—may provide a more robust and sustained protective effect in human HSPCs. Our in vivo data indicate that transient cAMP activation can attenuate the immediate apoptotic response of human hematopoietic cells to irradiation, independent of measurable changes in donor-derived IFNγ or TNFα. This points to a direct effect on intrinsic apoptosis pathways, although the impact on the host microenvironment requires further investigation. Indeed, the lack of improvement in secondary repopulation suggests that IR-induced persistent DNA damage signaling, oxidative stress, and radiation-induced bystander effects - still undermines long-term HSC function [36, 73]. These broader lesions likely lie beyond the protective reach of short-term cAMP elevation alone. Incorporating additional agents that mitigate oxidative damage or preserve the stromal microenvironment may therefore be required to achieve durable radioprotection in vivo.

In conclusion, our integrated molecular and functional analyses establish that activation of cAMP/CREB signaling pathway strongly potentiates human HSPCs regenerative abilities after DNA damage via decreasing ASPP1/p53 transcriptional activity and preserving vital MCL1 and BCL-XL protein levels. We predict that focusing on the pharmacological modulation of ASPP1/p53 axis in primary human HSPCs may be more directly relevant for therapeutic strategies aimed to mitigate DNA damage associated myelosuppression and genome editing efficacy without promoting transformation.

## Supplementary information

Supplemental methods and figure legends

Supplemental Figure 1

Supplemental Figure 2

Supplemental Figure 3

Supplemental Figure 4

Supplemental Figure 5

Supplemental Figure 6

Supplemental Figure 7

Supplemental Figure 8

Supplemental Figure 9

Supplemental Figure 10

Supplemental Figure 11

Supplemental Figure 12

Supplemental Figure 13

Supplemental Figure 14

Supplemental Figure 15

Supplemental Figure 16

## Data Availability

All raw sequencing data generated in this study have been submitted to the NCBI Gene Expression Omnibus (GEO; https://www.ncbi.nlm.nih.gov/geo/) under the accession number GSE247652.

## References

[1] Kaushansky K. Lineage-Specific Hematopoietic Growth Factors. N Engl J Med. 2006;354:2034–45. 16687716 10.1056/NEJMra052706

[2] Doulatov S, Notta F, Laurenti E, Dick JE. Hematopoiesis: A human perspective. Cell Stem Cell. 2012;10:120–36. 22305562 10.1016/j.stem.2012.01.006

[3] Niedernhofer LJ. DNA repair is crucial for maintaining hematopoietic stem cell function. DNA Repair. 2008;7:523–9. 18248857 10.1016/j.dnarep.2007.11.012 PMC2731414

[4] Milyavsky M, Gan OI, Trottier M, Komosa M, Tabach O, Notta F, et al. A Distinctive DNA damage response in human hematopoietic stem cells reveals an apoptosis-independent role for p53 in self-renewal. Cell Stem Cell. 2010;7:186–97. 20619763 10.1016/j.stem.2010.05.016

[5] Biechonski S, Olender L, Zipin-Roitman A, Yassin M, Aqaqe N, Marcu-Malina V, et al. Attenuated DNA damage responses and increased apoptosis characterize human hematopoietic stem cells exposed to irradiation. Sci Rep. 2018;8:1–13. 29666389 10.1038/s41598-018-24440-w PMC5904119

[6] Shao L, Sun Y, Zhang Z, Feng W, Gao Y, Cai Z, et al. Deletion of proapoptotic Puma selectively protects hematopoietic stem and progenitor cells against high-dose radiation. Blood. 2010;115:4707–14. 20360471 10.1182/blood-2009-10-248872 PMC2890171

[7] Henry E, Souissi-Sahraoui I, Deynoux M, Lefèvre A, Barroca V, Campalans A, et al. Human hematopoietic stem/progenitor cells display reactive oxygen species-dependent long-term hematopoietic defects after exposure to low doses of ionizing radiations. Haematologica. 2020;105:2044–55. 31780635 10.3324/haematol.2019.226936 PMC7395291

[8] Bohler S, Afreen S, Fernandez-Orth J, Demmerath EM, Molnar C, Wu Y, et al. Inhibition of the anti-apoptotic protein MCL-1 severely suppresses human hematopoiesis. Haematologica. 2021;106:3136–48. 33241675 10.3324/haematol.2020.252130 PMC8634190

[9] Yamashita M, Nitta E, Suda T. Aspp1 Preserves Hematopoietic Stem Cell Pool Integrity and Prevents Malignant Transformation. Cell Stem Cell. 2015;17:23–34. 26119234 10.1016/j.stem.2015.05.013

[10] Fuld S, Borland G, Yarwood SJ. Elevation of cyclic AMP in Jurkat T-cells provokes distinct transcriptional responses through the protein kinase A (PKA) and exchange protein activated by cyclic AMP (EPAC) pathways. Exp Cell Res. 2005;309:161–73. 15963501 10.1016/j.yexcr.2005.05.016

[11] Zhou BO, Yu H, Yue R, Zhao Z, Rios JJ, Naveiras O, et al. Bone marrow adipocytes promote the regeneration of stem cells and haematopoiesis by secreting SCF. Nat Cell Biol. 2017;19:891–903. 28714970 10.1038/ncb3570 PMC5536858

[12] Mourcin F, Grenier N, Mayol JF, Lataillade JJ, Sotto JJ, Hérodin F, et al. Mesenchymal stem cells support expansion of in vitro irradiated CD34 + cells in the presence of SCF, FLT3 ligand, TPO and IL3: Potential application to autologous cell therapy in accidentally irradiated victims. Radiat Res. 2005;164:1–9. 15966759 10.1667/rr3384

[13] Doan PL, Himburg HA, Helms K, Russell JL, Fixsen E, Quarmyne M, et al. Epidermal growth factor regulates hematopoietic regeneration after radiation injury. Nat Med. 2013;19:295–304. 23377280 10.1038/nm.3070 PMC3594347

[14] Bjarnadóttir TK, Fredriksson R, Schiöth HB. The Adhesion GPCRs: A unique family of G protein-coupled receptors with important roles in both central and peripheral tissues. Cellular and Molecular Life Sciences. 2007;64:2104–19. 17502995 10.1007/s00018-007-7067-1 PMC11138446

[15] Katoh O, Takahashi T, Oguri T, Kuramoto K, Mihara K, Kobayashi M, et al. Vascular endothelial growth factor inhibits apoptotic death in hematopoietic cells after exposure to chemotherapeutic drugs by inducing MCL1 acting as an antiapoptotic factor. Cancer Res. 1998;58:5565–9. 9850095

[16] Arai F, Hirao A, Ohmura M, Sato H, Matsuoka S, Takubo K, et al. Tie2/angiopoietin-1 signaling regulates hematopoietic stem cell quiescence in the bone marrow niche. Cell. 2004;118:149–61. 15260986 10.1016/j.cell.2004.07.004

[17] Himburg HA, Yan X, Doan PL, Quarmyne M, Micewicz E, McBride W, et al. Pleiotrophin mediates hematopoietic regeneration via activation of RAS. Journal of Clinical Investigation. 2014;124:4753–8. 25250571 10.1172/JCI76838 PMC4347246

[18] Himburg HA, Muramoto GG, Daher P, Meadows SK, Russell JL, Doan P, et al. Pleiotrophin regulates the expansion and regeneration of hematopoietic stem cells. Nat Med. 2010;16:475–82. 20305662 10.1038/nm.2119 PMC3689427

[19] Himburg HA, Termini CM, Schlussel L, Kan J, Li M, Zhao L, et al. Distinct Bone Marrow Sources of Pleiotrophin Control Hematopoietic Stem Cell Maintenance and Regeneration. Nat Commun. 2013;23:1–17. 10.1016/j.stem.2018.07.003 PMC6482945 30100167

[20] Himburg HA, Doan PL, Quarmyne M, Yan X, Sasine J, Zhao L, et al. Dickkopf-1 promotes hematopoietic regeneration via direct and niche-mediated mechanisms. Nat Med. 2017;23:91–9. 27918563 10.1038/nm.4251 PMC5592969

[21] Goncalves KA, Silberstein L, Li S, Severe N, Hu MG, Yang H, et al. Angiogenin Promotes Hematopoietic Regeneration by Dichotomously Regulating Quiescence of Stem and Progenitor Cells. Cell. 2016;166:894–906. 27518564 10.1016/j.cell.2016.06.042 PMC4988404

[22] Zhao M, Perry JM, Marshall H, Venkatraman A, Qian P, He XC, et al. Megakaryocytes maintain homeostatic quiescence and promote post-injury regeneration of hematopoietic stem cells. Nat Med. 2014;20:1321–6. 25326798 10.1038/nm.3706

[23] Zhao M, Ross JT, Itkin T, Perry JM, Venkatraman A, Haug JS, et al. FGF signaling facilitates postinjury recovery of mouse hematopoietic system. Blood. 2012;120:1831–42. 22802336 10.1182/blood-2011-11-393991 PMC3433089

[24] Ludin A, Itkin T, Gur-Cohen S, Mildner A, Shezen E, Golan K, et al. Monocytes-macrophages that express α-smooth muscle actin preserve primitive hematopoietic cells in the bone marrow. Nat Immunol. 2012;13:1072–82. 22983360 10.1038/ni.2408

[25] Demmerath EM, Bohler S, Kunze M, Erlacher M. In vitro and in vivo evaluation of possible pro-survival activities of PGE2, EGF, TPO and FLT3L on human hematopoiesis. Haematologica. 2019;104:669–77. 30442724 10.3324/haematol.2018.191569 PMC6442978

[26] Van Bekkum DW. Radiation sensitivity of the hemopoietic stem cell. Radiat Res. 1991;128:8–13. 1924746

[27] Ghandhi SA, Smilenov L, Shuryak I, Pujol-Canadell M, Amundson SA. Discordant gene responses to radiation in humans and mice and the role of hematopoietically humanized mice in the search for radiation biomarkers. Sci Rep. 2019;9:1–13. 31857640 10.1038/s41598-019-55982-2 PMC6923394

[28] Lane AA, Scadden DT. Stem cells and DNA damage: Persist or perish?. Cell. 2010;142:360–2. 20691895 10.1016/j.cell.2010.07.030

[29] Parekh C, Crooks GM. Critical differences in hematopoiesis and lymphoid development between humans and mice. J Clin Immunol. 2013;33:711–5. 23274800 10.1007/s10875-012-9844-3 PMC3633618

[30] Rangarajan A, Hong SJ, Gifford A, Weinberg RA, Parekh C, Crooks GM. Erratum to Species- and Cell Type-Specific Requirements for Cellular Transformation. [Cancer Cell. 2004;6:171–183. J Clin Immunol. 2013;24:394–8. 10.1016/j.ccr.2004.07.009 15324700

[31] Köhler A, Schmithorst V, Filippi MD, Ryan MA, Daria D, Gunzer M, et al. Niches for Hematopoietic Stem Cells and Their Progeny. Blood. 2020;20:833–46.

[32] Hurwitz SN, Jung SK, Kurre P. Hematopoietic stem and progenitor cell signaling in the niche. Leukemia. 2020;34:3136–48. 33077865 10.1038/s41375-020-01062-8

[33] Méndez-Ferrer S, Michurina TV, Ferraro F, Mazloom AR, MacArthur BD, Lira SA, et al. Mesenchymal and haematopoietic stem cells form a unique bone marrow niche. Nature. 2010;466:829–34. 20703299 10.1038/nature09262 PMC3146551

[34] Greenbaum A, Hsu YMS, Day RB, Schuettpelz LG, Christopher MJ, Borgerding JN, et al. CXCL12 in early mesenchymal progenitors is required for haematopoietic stem-cell maintenance. Nature. 2013;495:227–30. 23434756 10.1038/nature11926 PMC3600148

[35] Diaz MF, Horton P, Lina D, Dumbali SP, Kumar A, Livingston M, Skibber MA, et al. Bone marrow stromal cell therapy improves survival after radiation injury but does not restore endogenous hematopoiesis. Sci Rep. 2020;10:1–19. 33335275 10.1038/s41598-020-79278-y PMC7747726

[36] Abbuehl JP, Tatarova Z, Held W, Huelsken J. Long-Term Engraftment of Primary Bone Marrow Stromal Cells Repairs Niche Damage and Improves Hematopoietic Stem Cell Transplantation. Cell Stem Cell [Internet]. 2017;21:241–55.e6. 28777945 10.1016/j.stem.2017.07.004

[37] Yang SJ, Wang XQ, Jia YH, Wang R, Cao K, Zhang X, et al. Human umbilical cord mesenchymal stem cell transplantation restores hematopoiesis in acute radiation disease. Am J Transl Res. 2021;13:8670–82. 34539986 PMC8430114

[38] Drouet M, Mourcin F, Grenier N, Delaunay C, Mayol JF, Lataillade JJ, et al. Mesenchymal stem cells rescue CD34+ cells from radiation-induced apoptosis and sustain hematopoietic reconstitution after coculture and cografting in lethally irradiated baboons: Is autologous stem cell therapy in nuclear accident settings hype or reality. Bone Marrow Transplant. 2005;35:1201–9. 15821761 10.1038/sj.bmt.1704970

[39] Liang H, Ao Y, Li W, Liang K, Tang B, Li J, et al. Injectable bone marrow microniches by co-culture of HSPCs with MSCs in 3D microscaffolds promote hematopoietic reconstitution from acute lethal radiation. Bioact Mater. 2023;22:453–65. 36311043 10.1016/j.bioactmat.2022.10.015 PMC9593104

[40] Hu KX, Sun QY, Guo M, Ai HS. The radiation protection and therapy effects of mesenchymal stem cells in mice with acute radiation injury. British Journal of Radiology. 2010;83:52–8. 20139249 10.1259/bjr/61042310 PMC3487250

[41] Shim S, Lee SB, Lee JG, Jang WS, Lee SJ, Park S, et al. Mitigating effects of hUCB-MSCs on the hematopoietic syndrome resulting from total body irradiation. Exp Hematol. 2013;41:346–53.e2. 23333483 10.1016/j.exphem.2013.01.002

[42] Chinnadurai R, Forsberg MH, Kink JA, Hematti P, Capitini CM. Use of MSCs and MSC-Educated Macrophages to Mitigate Hematopoietic Acute Radiation Syndrome. Curr Stem Cell Rep. 2020;6:77–85. 32944493 10.1007/s40778-020-00176-0 PMC7491866

[43] Wen S, Dooner M, Cheng Y, Papa E, Del Tatto M, Pereira M, et al. Mesenchymal stromal cell-derived extracellular vesicles rescue radiation damage to murine marrow hematopoietic cells. Leukemia. 2016;30:2221–31. 27150009 10.1038/leu.2016.107 PMC5093052

[44] Porter RL, Georger MA, Bromberg O, Mcgrath KE, Frisch BJ, Becker MW, et al. Prostaglandin E2 increases hematopoietic stem cell survival and accelerates hematopoietic recovery after radiation injury. Stem Cells. 2013;31:372–83. 23169593 10.1002/stem.1286 PMC3580384

[45] Wang Y, Lai S, Tang J, Feng C, Liu F, Su C, et al. Prostaglandin E2 promotes human CD34+ cells homing through EP2 and EP4 in vitro. Mol Med Rep. 2017;16:639–46. 28560401 10.3892/mmr.2017.6649 PMC5482140

[46] Ikushima YM, Arai F, Hosokawa K, Toyama H, Takubo K, Furuyashiki T, et al. Prostaglandin E2 regulates murine hematopoietic stem/progenitor cells directly via EP4 receptor and indirectly through mesenchymal progenitor cells. Blood. 2013;121:1995–2007. 23315170 10.1182/blood-2012-06-437889

[47] Sporrij A, Choudhuri A, Prasad M, Muhire B, Fast EM, Manning ME, et al. PGE2 alters chromatin through H2A.Z-variant enhancer nucleosome modification to promote hematopoietic stem cell fate. Proc Natl Acad Sci USA. 2023;120. 10.1073/pnas.2220613120 PMC10175842 37126722

[48] Goessling W, Allen RS, Guan X, Jin P, Uchida N, Dovey M, et al. Prostaglandin E2 enhances human cord blood stem cell xenotransplants and shows long-term safety in preclinical nonhuman primate transplant models. Cell Stem Cell. 2011;8:445–58. 21474107 10.1016/j.stem.2011.02.003 PMC3148081

[49] Cutler C, Multani P, Robbins D, Kim HT, Le T, Hoggatt J, et al. Prostaglandin-modulated Umbilical cord blood hematopoietic stem cell transplantation. Blood. 2013;122:3074–81. 23996087 10.1182/blood-2013-05-503177 PMC3811179

[50] Goichberg P, Kalinkovich A, Borodovsky N, Tesio M, Petit I, Nagler A, et al. cAMP-induced PKCδ activation increases functional CXCR4 expression on human CD34+ hematopoietic progenitors. Blood. 2006;107:870–9. 16204315 10.1182/blood-2005-03-0941

[51] Poletti V, Montepeloso A, Pellin D, Biffi A. Prostaglandin E2 as transduction enhancer affects competitive engraftment of human hematopoietic stem and progenitor cells. Mol Ther Methods Clin Dev. 2023;31:101131. 37920236 10.1016/j.omtm.2023.101131 PMC10618226

[52] Heffner GC, Bonner M, Christiansen L, Pierciey FJ, Campbell D, Smurnyy Y, et al. Prostaglandin E2 Increases Lentiviral Vector Transduction Efficiency of Adult Human Hematopoietic Stem and Progenitor Cells. Molecular Therapy. 2018;26:320–8. 29102562 10.1016/j.ymthe.2017.09.025 PMC5763075

[53] Zonari E, Desantis G, Petrillo C, Boccalatte FE, Lidonnici MR, Kajaste-Rudnitski A, et al. Efficient Ex Vivo Engineering and Expansion of Highly Purified Human Hematopoietic Stem and Progenitor Cell Populations for Gene Therapy. Stem Cell Reports. 2017;8:977–90. 28330619 10.1016/j.stemcr.2017.02.010 PMC5390102

[54] Patterson AM, Liu L, Sampson CH, Plett PA, Li H, Singh P, et al. A Single Radioprotective Dose of Prostaglandin E2 Blocks Irradiation-Induced Apoptotic Signaling and Early Cycling of Hematopoietic Stem Cells. Stem Cell Reports. 2020;15:358–73. 32735825 10.1016/j.stemcr.2020.07.004 PMC7419738

[55] Gao J, Yan XL, Li R, Liu Y, He W, Sun S, et al. Characterization of OP9 as authentic mesenchymal stem cell line. Journal of Genetics and Genomics. 2010;37:475–82. 20659712 10.1016/S1673-8527(09)60067-9

[56] Magnusson M, Sierra MI, Sasidharan R, Prashad SL, Romero M, Saarikoski P, et al. Expansion on Stromal Cells Preserves the Undifferentiated State of Human Hematopoietic Stem Cells Despite Compromised Reconstitution Ability. PLoS One. 2013;8. 10.1371/journal.pone.0053912 PMC3547050 23342037

[57] Shu M, Zhang J, Peng Y, Li Z, Shu X, Wang J, et al. Programmed cell death regulates hematopoietic cell homeostasis under radiation conditions. Stem Cell Res Ther [Internet]. [cited 2025 Dec 2];16:390. Available from: (2025) https://pmc.ncbi.nlm.nih.gov/articles/PMC12281764/. 10.1186/s13287-025-04502-3 PMC12281764 40691803

[58] Hoggatt J, Singh P, Sampath J, Pelus LM. Prostaglandin E2 enhances hematopoietic stem cell homing, survival, and proliferation. Blood. 2009;113:5444–55. 19324903 10.1182/blood-2009-01-201335 PMC2689046

[59] Johnson CS, Williams M, Sham K, Belluschi S, Ma W, Wang X, et al. Adaptation to ex vivo culture reduces human hematopoietic stem cell activity independently of the cell cycle. Blood. 2024;144:729–41. 38805639 10.1182/blood.2023021426 PMC7616366

[60] Moschoi R, Imbert V, Nebout M, Chiche J, Mary D, Prebet T, et al. Protective mitochondrial transfer from bone marrow stromal cells to acute myeloid leukemic cells during chemotherapy. Blood. 2016;128:253–64. 27257182 10.1182/blood-2015-07-655860

[61] Liao W, Liu C, Yang K, Chen J, Wu Y, Zhang S, et al. Aged hematopoietic stem cells entrap regulatory T cells to create a prosurvival microenvironment. Cell Mol Immunol. 2023;20:1216–31. 37644165 10.1038/s41423-023-01072-3 PMC10541885

[62] Yu H, Shen H, Yuan Y, XuFeng R, Hu X, Garrison SP, et al. Deletion of Puma protects hematopoietic stem cells and confers long-term survival in response to high-dose γ-irradiation. Blood. 2010;115:3472–80. 20177048 10.1182/blood-2009-10-248278 PMC2867261

[63] Liu L, Li H, Patterson AM, Plett PA, Sampson CH, Mohammad KS, et al. Upregulation of SIRT1 Contributes to dmPGE2-dependent Radioprotection of Hematopoietic Stem Cells. Stem Cell Rev Rep. 2022;18:1478–94. 35318613 10.1007/s12015-022-10368-2

[64] Goodman RH, Smolik S. CBP/p300 in cell growth, transformation, and development. Genes Dev. 2000;14:1553–77. 10887150

[65] Dyson HJ, Wright PE. Role of intrinsic protein disorder in the function and interactions of the transcriptional coactivators CREB-binding Protein (CBP) and p300. Journal of Biological Chemistry. 2016;291:6714–22. 26851278 10.1074/jbc.R115.692020 PMC4807259

[66] Deverman BE, Cook BL, Manson SR, Niederhoff RA, Langer EM, Rosová I, et al. Bcl-xL deamidation is a critical switch in the regulation of the response to DNA damage. Cell. 2002;111:51–62. 12372300 10.1016/s0092-8674(02)00972-8

[67] Dho SH, Deverman BE, Lapid C, Manson SR, Gan L, Riehm JJ, et al. Control of Cellular Bcl-xL Levels by Deamidation-Regulated Degradation. PLoS Biol. 2013;11:e1001588. 23823868 10.1371/journal.pbio.1001588 PMC3692414

[68] Afreen S, Bohler S, Müller A, Demmerath EM, Weiss JM, Jutzi JS, et al. BCL-XL expression is essential for human erythropoiesis and engraftment of hematopoietic stem cells. Cell Death Dis. 2020;11:8. 31907357 10.1038/s41419-019-2203-z PMC6944703

[69] Opferman JT, Iwasaki H, Ong CC, Suh H, Mizuno SI, Akashi K, et al. Obligate role of anti-apoptotic MCL-1 in the survival of hematopoietic stem cells. Science. 2005;307:1101–4. 15718471 10.1126/science.1106114

[70] Christophorou MA, Ringshausen I, Finch AJ, Swigart LB, Evan GI. The pathological response to DNA damage does not contribute to p53-mediated tumour suppression. Nature. 2006;443:214–7. 16957739 10.1038/nature05077

[71] Michalak EM, Vandenberg CJ, Delbridge ARD, Wu L, Scott CL, Adams JM, et al. Apoptosis-promoted tumorigenesis: γ-irradiation-induced thymic lymphomagenesis requires Puma-driven leukocyte death. Genes Dev. 2010;24:1608–13. 20679396 10.1101/gad.1940110 PMC2912558

[72] Labi V, Erlacher M, Krumschnabel G, Manzl C, Tzankov A, Pinon J, et al. Apoptosis of leukocytes triggered by acute DNA damage promotes lymphoma formation. Genes Dev. 2010;24:1602–7. 20679395 10.1101/gad.1940210 PMC2912557

[73] Hu L, Yin X, Zhang Y, Pang A, Xie X, Yang S, et al. Radiation-induced bystander effects impair transplanted human hematopoietic stem cells via oxidative DNA damage. Blood. 2021;137:3339–50. 33881475 10.1182/blood.2020007362 PMC8233686

[74] Kulesza A, Paczek L, Burdzinska A. The Role of COX-2 and PGE2 in the Regulation of Immunomodulation and Other Functions of Mesenchymal Stromal Cells. Biomedicines. 2023;11:445. 36830980 10.3390/biomedicines11020445 PMC9952951

[75] de Oliveira Bravo M, Carvalho JL, Saldanha-Araujo F. Adenosine production: a common path for mesenchymal stem-cell and regulatory T-cell-mediated immunosuppression. Vol. 12, Purinergic Signalling. Springer Netherlands; 2016. p. 595–609. 10.1007/s11302-016-9529-0 PMC5124006 27557887

[76] Ahlström M, Pekkinen M, Lamberg-Allardt C. Dexamethasone downregulates the expression of parathyroid hormone-related protein (PTHrP) in mesenchymal stem cells. Steroids. 2009;74:277–82. 19121329 10.1016/j.steroids.2008.12.002

