# Supplementary material for: Mesenchymal stromal cells modulate survival and regeneration of human hematopoietic stem cells via PGE2/cAMP signaling
Source: Cell Death Dis. 2026 Mar 3;17(1):307. doi: 10.1038/s41419-026-08502-w (PMC13039678; doi:10.1038/s41419-026-08502-w)
Supplement: Supplementary file 1 — Supplemental methods and figure legends [file 41419_2026_8502_MOESM1_ESM.docx]

**Materials and Methods**

**CD34+ HSPCs purification**

Cord blood units and bone marrow samples were obtained according to procedures approved by the institutional review boards of the Sheba Medical Centre, Israel and Tel Aviv University. Informed consent was obtained from all subjects. Two-six deidentified cord blood units were pooled, and mononuclear cells were isolated by density gradient centrifugation. CD34+ cells from cord blood or from the bone marrow samples were enriched by positive selection with MACS CD34+ ultra-pure kit (Miltenyi Biotech, Cat# 130-100-453) according to the manufacturer’s instructions. Purified cells were stored in liquid nitrogen and used at later time points.

**CD34+ HSPCs culture**

Thawed CD34+ cells were cultured in StemSpan SFEM II serum-free medium (Stem cell technologies, Cat# 9655) supplemented with recombinant cytokines (Peprotech): SCF (25ng/ml), FLT3L (25ng/ml), TPO (25ng/ml). To elevate cAMP levels, CD34+ cells were treated with Forskolin (10μM, Alomone labs Cat# F-500) and IBMX (100μM, Sigma Aldrich). CD34+ cells were treated with following agents– Etoposide (1µM, Sigma Aldrich Cat# I5879), Rp-8-Br-cAMPs (100µM, BioLog Cat# B001-05), Carbenoxolone (100µM, Sigma Aldrich Cat# C4790), GAP27 (100µM, Med Chem Express Cat# HY-P0139), 16,16-dmPGE2 (10µM, Cayman Chemical Cat# 14750), AH6809 (10µM, Med Chem Express Cat# HY-10418), Palupiprant (1µM, Med Chem Express Cat# HY-103088).

**CD34+ HSPCs and stroma co-culture**

OP9M2 murine stromal cells were cultured in MEM-Alpha medium (Gibco, Life Technologies Cat# 12571063), supplemented with FBS (20%, Biological Industries), Pen/Strep (1%), L-glutamine (1%). Omi-MS5 cells were generated by transducing MS5 cells with pBabe(puro)-Omi-mCherry plasmid (Addgene plasmid# 48685). MS5 and Omi-MS5 cells were cultured in MEM-Alpha medium (Gibco, Life Technologies), supplemented with FBS (10%, GIBCO Cat# 04-007-1A), Pen/Strep (1%) (Diagvonum Cat# D910), L-glutamine (1%) (GIBCO Cat# 289081).

Bone marrow derived MSCs were isolated from healthy donors undergoing hip replacement surgery under Meir Medical Center Helsinki Committee approval and informed consent. MSCs were expanded in RPMI-1640 medium supplemented with 10 % FBS and standard antibiotics, and used between passages 3-5. MSCs were phenotypically validated as CD271⁺CD34⁻CD45⁻/vimentin⁺/keratin⁻ as previously described.

For co-culture with CD34+ cells, 3*10^4^ OP9M2 cells or 5*10^4^ MS5 cells were plated in 24-well tissue culture treated plate (Greiner Bio-One Cat# 662160) and incubated in the stroma medium (see above) for 24 hours. Following the incubation, medium was aspirated, and CD34+ enriched cells were plated on MSCs in StemSpan SFEM II serum-free medium supplemented with cytokines. Cells were maintained in a humidified incubator at 37 °C and CO2 (5%).

**Annexin V apoptosis assay**

To induce apoptosis, CD34+ cells cultured in different conditions were irradiated at 3 Gy using a Biobeam gamma-irradiator (Gamma service) or alternatively allowed to cycle for 72 hours before irradiation. For treatment with Etoposide, CD34+ cells were cultured for 24 hours and then treated with Etoposide (1µM) for an additional 48 hours. CD34+ cells were then washed and incubated for 30 mins with antibodies for surface markers – CD45RA BV605 (1:200), CD38 PE/Cy7 (1:100), and CD34 PE (1:100). CD34+ cells were then washed and stained with Annexin V- AF488 (Invitrogen) and Sytox blue dead cell stain (Invitrogen). Cells were analysed with Cytoflex flow cytometer (Beckman Coulter).

**Intracellular flow cytometry**

CD34+ cells were cultured in different conditions as indicated and stained with antibodies for HSPC surface markers - CD45RA BV605, CD38 PE/Cy7 and CD34 PE. Zombie NIR dye (Biolegend) was used to label dead cells. Cells were then fixed with 1.6% Paraformaldehyde and permeabilized in 90% ethanol followed by labelling with AF488-conjugated intracellular antibodies for BCL-2, BCL-XL, MCL-1, P-CREB-Ser133 and Survivin.

**Mitochondrial mass & Mitochondrial Membrane Potential analysis**

CD34+ cells cultured in different conditions were treated with Verapamil (50μM, Sigma Cat# V4629) for 30 mins. MitoTracker Green FM (400nM, Cell Signaling Technology) or TMRE (200nM, Abcam) were added for additional 30 mins. Cells were washed twice, stained with cell surface markers and analyzed by flow cytometry.

**NOD/SCID repopulating cell assay**

All animal experimental protocols were approved by the Institutional Animal Care and Use Committee of Tel-Aviv University, Israel (TAU-MD-IL-2307-152-5). Mice were housed within the Tel Aviv University Specific Pathogen Free (SPF) facility in individually ventilated cages with four to five animals of the same sex per cage. All mice were maintained on a regular diurnal lighting cycle (12:12 light: dark) with ad libitum access to food and water.

For primary transplantation, 100,000 human CD34+ cells (treated as indicated, irradiated at 3 Gy, and cultured for 24 h) were transplanted into female NSGW41 mice (8–12 weeks old). Mice were anesthetized with isoflurane (3% in 2.5% oxygen via a calibrated vaporizer), and a drop of ophthalmic lidocaine was applied to the eye immediately before injection. Cells were delivered intravenously into the retrobulbar plexus while animals remained under continuous isoflurane anesthesia via a nose cone.  To ensure humane endpoints, mice were monitored daily for health parameters according to the protocol approved by the Institutional Animal Care and Use Committee of Tel-Aviv University (TAU-MD-IL−2307−152–5). Changes in any of the following parameters were defined as a human endpoint: body weight loss of 20% or more between the measurements (monitored by laboratory balances), lethargy, ruffled fur, hind limb paralysis, hunched posture and labored breathing (all monitored by visual inspection). Upon meeting the humane endpoint criteria or twelve to fifteen weeks after the injection, mice were euthanized by CO2 inhalation using a fill rate of 50% of the chamber volume/minute. CO2 flow was maintained for at least 2 min after breathing stops to ensure death. Euthanasia was confirmed by the absence of breathing and reflexes. No secondary method of euthanasia was performed. Bone Marrow, Spleen and Peripheral Blood engraftment was analyzed by flow cytometry using hCD45, hCD33, hCD19 and hCD3 antibodies.

For secondary transplantation, 90% of whole bone marrow of primary mice (> 1% hCD45 chimerism) was reinjected into secondary NSGW41 mice intravenously into the retrobulbar plexus and the BM engraftment was analyzed by immunostaining and flow cytometry after additional 12 weeks.

**In vivo protection assay in engrafted human hematopoietic cells**

NSG mice (8–12 weeks) were busulfan-conditioned (25 mg/kg, i.p.) 24 h before retro-orbital transplantation of 50,000 human cord blood CD34+ cells. After 12 weeks, engrafted mice (based on peripheral blood human CD45+ chimerism) were randomized into three groups: 1) DMSO vehicle, 2) DMSO + 3 Gy total body irradiation (TBI), or 3) Forskolin (10 mg/kg) + IBMX (2 mg/kg) i.p. 1 h before TBI. Bone marrow was collected 24 h later for flow cytometric analysis of human CD45+ chimerism and Annexin V/Sytox Blue staining to quantify apoptosis. Bone marrow fluid was collected to quantify human IFNγ and TNFα levels by ELISA.

To evaluate long-term functional protection, ~90% of the total bone marrow from each primary mouse was transplanted into secondary NSG recipients, and human hematopoietic chimerism was analysed 12 weeks later.

**In Vitro Limiting dilution analysis**

96 well plates (Greiner Bio-One) were covered with 0.1% gelatin solution (Biological Industries) for 20 minutes, and dried open in a sterile hood for 120 minutes. Then, coated wells were seeded with MS5 stromal cells (7000 cells/ well) in 100μl of H5100 medium (STEM CELL technologies) supplemented with 1μM hydrocortisone (Sigma) + 1% P/S. MS-5 cells were irradiated (20 Gy) after seeding into 96 well plates. Twenty-four hours later 10/50/500 non irradiated CD34^+^ cells or 1,500/7,500/15,000 irradiated (3Gy) CD34^+^ cells per well were plated in 100 μl MyeloCult H5100 medium (STEM CELL technologies). Once a week half medium replacement was done. After 5 weeks the H5100 medium was replaced with Methocult H4434 Classic methylcellulose medium (100µl/ well, STEM CELL technologies). Wells that contained at least one colony after 10-14 days were considered positive.

**RNA sequencing**

CD34+ cells were cultured in SFT medium or on MSCs for 24 hours, were stained for HSPCs markers and sorted using BD FACS AriaIII cytometer. Total RNA was extracted using TRIZOL reagent (Invitrogen, Cat# 15596018) and quality tested using a 2100 Bioanalyzer (Agilent technologies). Sequencing Libraries were prepared using MARSeq. Single reads were sequenced on 1 lane(s) of an Illumina NOvaSeq_sp. The output was ~15 million reads per sample.

**Data analysis**

Poly-A/T stretches and Illumina adapters were trimmed from the reads using cutadapt^1^; resulting reads shorter than 30bp were discarded. Reads were mapped to the H. sapiens reference genome GRCh38 using STAR^2^, supplied with gene annotations downloaded from Ensembl (and with EndToEnd option and outFilterMismatchNoverLmax was set to 0.04). Expression levels for each gene were quantified using htseq-count^3^, using the gtf above. Differentially expressed genes were identified using DESeq2^4^ with the betaPrior, cooksCutoff and independent filtering parameters set to False. Raw P values were adjusted for multiple testing using the procedure of Benjamini and Hochberg. The Pipeline was run using snakemake workflow engine^5^.

Differentially expressed (DE) genes from each comparison were mapped to promoter regions (−400 to +100 bp relative to the annotated TSS, Ensembl GRCh38), yielding BED files used as input. Motif enrichment was performed with HOMER (findMotifsGenome.pl, hg38; -size given), using HOMER’s default length/GC-matched genomic background. Enrichment statistics and fold enrichments were reported from HOMER’s known transcription-factor motif library; where indicated, analyses were restricted to a focused ATF/CREB/AP-1 motif set supplied via -mknown, and motif instances were enumerated with -find^6^.

**Gene Set Enrichment Analysis**

Gene Set Enrichment Analysis (GSEA)^7^ was done using the GSEA desktop application (Broad Institute). Genes were ranked by the DESeq2 statistic and pre-ranked GSEA was run using the GSEA hallmarks, Transcription Factor Targets (TFT) and other curated gene HSC gene sets from MSigDB database using standard settings. Heatmaps were created using Morpheus (Broad Institute).

**PGE2 detection by ELISA**

Conditioned medium from CD34+, OP9M2, MS5, hBM-MSCs or CD34+ - OP9M2 co-cultures were collected 24 hours after treatment.  PGE2 levels in the culture medium were determined by PGE2 Enzyme Immunoassay kit according to the manufacturer’s instructions (Cayman Chemicals, Cat# 514010-96).

**qRT-PCR analysis**

Total RNA was extracted using the RNeasy Micro kit (Qiagen Cat# 74004). cDNA synthesis was performed using the qScript cDNA Synthesis kit (Quanta Bio Cat# 95047-025). Real-time quantitative PCR was performed using PerfeCTa SYBR green supermix reagent (Quanta Bio Cat# 95054-500) and analysed by QuantStudio 5 Real-time PCR system (ThermoFisher). Relative expression was calculated for each gene using by 2^-ΔΔCT method. *GAPDH* was used for normalization.

**siRNA Nucleofection**

Dharmacon ON-TARGETplus siRNA pool oligos for non-targeting control, BCL2, BCL-XL and MCL1 were ordered. For electroporation, human 2.5 × 10^5^ CD34^+^ cells/100 µl were resuspended in nucleofection buffer (P3 primary cell kit, Lonza) and were nucleofected with CD34^+^ program using Amaxa 4D nucleofector system (Lonza). Control, si-BCL2, si-BCL-XL, si-MCL1 were used at a final concentration of 2 μM.

**Statistical analysis**

Statistical analysis was performed with GraphPad Prism 9, using student’s t-test. Statistical significance is defined as p<0.05. Bars represent Mean ± Standard error of mean (SEM) of independent experiments. *P≤0.05, **P≤0.01, ***P≤0.001, ****P≤0.0001.

**Supplementary figure legends**

**Supplemental Figure 1. Characterization of MSCs and initial HSPC apoptotic responses.**

**(A)** Representative histograms illustrating the MSC marker expression pattern of OP9M2cells. Unstained (isotype control) cells are shown in grey, whereas antibody-stained cells are depicted in red. **(B)** Flow cytometric analysis of apoptosis in cord blood (CB) CD34+CD38-CD45RA- cells (HSPCs) cultured for 24h—either in cytokine-supplemented medium alone or on human BM-derived MSCs or murine OP9M2 cells—following 3 Gy irradiation, as assessed by Annexin V staining (n = 3). **(C)** Human BM-derived CD34+ cells were cultured under the indicated conditions for 24 hours and flow cytometric analysis of apoptosis in CD34+CD38-CD45RA- HSPCs using Annexin V staining (n = 3). **(D)** CB CD34⁺ cells were cultured for 24 hours in serum-free medium supplemented with SFT cytokines, then treated with 1 µM Etoposide for 48 hours. Apoptosis was assessed by Annexin V staining in CD34+CD38-CD45RA- HSPCs (n = 3). **(E)** Quantification of mitochondrial membrane potential in CD34+CD38-CD45RA- HSPCs, as measured by TMRE staining 24 hours after IR and co-culture with OP9M2 MSCs (MFI values). **(F)** CB CD34⁺ cells were cultured under the indicated conditions for up to 24 hours and stained with MitoTracker Green FM to assess mitochondrial mass (n=3). Data are mean ± SEM. Statistical significance was assessed by unpaired two-tailed Student’s *t*-test.

**Supplemental Figure 2. Analysis of mitochondrial transfer and apoptosis in HSPCs.**

**(A)** CB CD34⁺ cells were cultured under the indicated conditions for up to 24 hours and flow cytometric analysis of apoptosis in CD34+CD38-CD45RA- HSPCs by Annexin V staining (n = 3). **(B)** mCherry fluorescence confirming the genetic labelling of MS5 cell mitochondria. **(C)** Representative FACS plots showing mCherry fluorescence in HSPCs cultured alone or in co-culture with mCherry-labelled MS5 cells.

**Supplemental Figure 3. LTC-IC assay and in vivo hematopoietic reconstitution.**

**(A**) CB-derived CD34⁺ cells were exposed to 3 Gy IR before being cultured for 24 hours in either cytokine-only or MSC co-culture conditions. Cells were then plated for a limiting dilution LTC-IC assay. After five weeks, the medium was replaced with methylcellulose, and wells were scored for colony formation 10–14 days later. The table summarizes LTC-IC frequencies as calculated by L-CALC software. **(B)** Lineage distribution of human cells in the bone marrow of recipient mice. **(C)** Human CD45⁺ chimerism in the peripheral blood of NSGW41 mice 15 weeks post-transplant. **(D)** Human CD45⁺ chimerism in the spleen of NSGW41 mice 15 weeks post-transplant.

**Supplemental Figure 4. MSC exposure triggers CREB-dependent gene expression changes and enrichment of stemness-associated transcriptional programs in human HSPCs**

**(A**) Heatmap showing differentially expressed genes in human CD34⁺CD38⁻CD45RA⁻ HSPCs after 24 h co-culture with OP9M2 MSCs compared to cytokine-only controls. Each group represents the average gene-expression profile of four independent biological and technical replicates. **(B)** GSEA enrichment plot for TAX-CREB target genes in HSPCs co-cultured with OP9M2 MSCs versus cytokine-only culture. **(C)** Enrichment plots for published HSC stemness-associated gene sets, along with a heatmap of genes downregulated upon MSC co-culture. **(D)** Heatmap displaying the expression of leading-edge CREB1 target genes. **(E)** qRT-PCR analysis validating upregulation of CREB target genes in HSPCs co-cultured with hBM-MSCs compared to controls (n = 5). Data are mean ± SEM. Statistical significance was assessed by unpaired two-tailed Student’s *t*-test.

**Supplemental Figure 5. Gating strategy and Annexin V–based quantification of apoptotic HSPCs following irradiation and cAMP antagonist treatment.**

**(A)** Gating scheme for apoptosis analysis in human CD34+CD38-CD45RA- HSPCs **(B)** FACS plots showing Annexin V+ apoptotic HSPCs percentage in HSPCs after cAMP antagonist treatment 24 hrs after irradiation. **(C)** CB CD34+ cells were cultured under the indicated conditions for up to 24 hours and analysed for intracellular phospho-CREB (Ser133) levels. Bar graphs represent the MFI values of P-CREB in HSPCs at 24 hours.

**Supplemental Figure 6. Gating strategy and Annexin V–based quantification of apoptotic HSPCs following irradiation and gap junction inhibitors treatment.**

**(A)** FACS plots showing Annexin V+ apoptotic HSPCs percentage in HSPCs after Connexin 43 mimetic peptide treatment 24 hrs after irradiation. **(B)** FACS plots showing Annexin V+ apoptotic HSPCs percentage in HSPCs after gap junction inhibitor treatment 24 hrs after irradiation.

**Supplemental Figure 7. Gating strategy and Annexin V–based quantification of apoptotic HSPCs following irradiation and EP2 and EP4 antagonists treatment.**

**(A)** FACS plots showing Annexin V+ apoptotic HSPCs percentage in HSPCs after prostaglandin receptor antagonist treatment 24 hrs after irradiation.

**Supplemental Figure 8. Dual EP2/EP4 inhibition abolishes MSC-mediated protection of irradiated human HSPCs**

**(A–C)** CB CD34+ cells cultured in the presence of individual or combination of prostaglandin receptor antagonists for up to 24 hours and analysed for apoptosis in HSPCs using Annexin V staining (n = 3). **(D)** OP9M2 MSCs were cultured under the indicated conditions for 24 hours, and PGE2 concentrations in the supernatants were measured by ELISA (n = 3).

**Supplemental Figure 9. Gating strategy and Annexin V–based quantification of apoptotic HSPCs following irradiation and Forskolin/ IBMX treatment.**

**(A)** FACS plots showing Annexin V+ apoptotic HSPCs percentage in HSPCs after Forskolin/ IBMX treatment 24 hrs after irradiation.

**Supplemental Figure 10. Effects of Forskolin/IBMX on cAMP/CREB activation and apoptosis.**

**(A)** CB CD34+ cells were cultured under the indicated conditions for up to 24 hours and analysed for intracellular phospho-CREB (Ser133) levels in HSPCs (n = 3–4). **(B)** CB CD34+ cells were cultured for 24 hours under the indicated conditions and apoptosis was quantified in HSPCs by Annexin V staining (n = 4). **(C)** CB CD34+ cells were cultured for 24 hours in serum-free medium with SFT cytokines, then treated with 1 µM Etoposide plus 10 µM Forskolin/100 µM IBMX for 48 hours. Apoptosis was assessed in HSPCs by Annexin V staining (n = 3). **(D)** Quantitative analysis (MFI values) of TMRE staining in HSPCs 24 hours after IR and Forskolin/IBMX treatment. **(E)** Annexin V staining of apoptosis in cycling CB HSPCs (induced by 72h culture) following 24h exposure to the indicated conditions (n = 3). Data are mean ± SEM. Statistical significance was assessed by unpaired two-tailed Student’s *t*-test. **(F)** qRT-PCR analysis of CREB target genes in CD34+ cells treated with dmPGE2 or Forskolin/IBMX for 3 hours (n = 4).

**Supplemental Figure 11. In vivo hematopoietic reconstitution analysis.**

**(A)** Human chimerism in the BM of mice transplanted with control or dmPGE2 treated CD34+ HSPCs **(B)** Lineage distribution in the bone marrow of transplanted recipient mice. **(C)** Human chimerism in the spleens of mice transplanted with control or Forskolin/IBMX-treated hCD34⁺ cells (expressed as % hCD45⁺ cells). **(D)** Human chimerism in the spleens of secondary recipient mice following transplantation of bone marrow from primary recipients treated with control or Forskolin/IBMX-treated hCD34⁺ cells.

**Supplemental Figure 12. In vivo activation of cAMP signaling reduces irradiation-induced apoptosis in human hematopoietic cells.**

**(A)** Schematic of in vivo protection experiment. NSG mice were busulfan-conditioned and transplanted with 5 × 10⁴ human CB CD34+ cells. Twelve weeks later, mice were randomized to receive vehicle (DMSO), vehicle + 3 Gy TBI, or Forskolin (10 mg/kg) + IBMX (2 mg/kg) 1 h before 3 Gy TBI. **(B)** Percentage of human CD45⁺ cells in bone marrow 24h after irradiation. **(C)** Percentage of apoptotic (Annexin V+) human CD45+ cells in bone marrow. **(D-E)** Human IFNγ and TNFα levels in the mouse BM fluid was quantified 24 hours after Total Body Irradiation. **(F-G)** Human chimerism in the BM and spleens of secondary recipient mice following transplantation of bone marrow from primary recipients.

**Supplemental Figure 13. qRT-PCR analysis of pro-survival BCL-2 family gene expression.**

CB CD34⁺ cells were treated with dmPGE2 or Forskolin/IBMX, and gene expression was quantified by qRT-PCR at different time points: **(A)** BCL2 at 3 hours; **(B)** BCL-XL at 3 hours; **(C)** MCL1 at 3 hours; **(D)** BCL2 at 20 hours; **(E)** BCL-XL at 20 hours; **(F)** MCL1 at 20 hours. CB CD34+ cells were nucleofected with siRNAs against **(G)** BCL2 **(H)** BCL-XL **(I)** MCL1 and culture for 24 hrs. Gene knockdown efficiency was quantified by qRT-PCR.

**Supplemental Figure 14. Flow cytometric quantification of anti-apoptotic proteins post-irradiation.**

CB-derived CD34⁺ cells were cultured for 24 hours after irradiation in SFEM medium with cytokines or in co-culture with OP9M2 MSCs. Intracellular protein levels in HSPCs were measured by flow cytometry: **(A)** BCL2; **(B)** BCL-XL; **(C)** MCL1. Left panels show representative histograms; right panels depict relative expression changes, expressed as the ratio of MFI between IR and non-IR conditions (n = 4 for BCL2 and BCL-XL; n = 6 for MCL1).

**Supplemental Figure 15. Analysis of intracellular anti-apoptotic protein levels following Forskolin/IBMX treatment.**

CB-derived CD34⁺ cells were cultured for 24 hours after irradiation in SFEM medium with cytokines, with or without Forskolin/IBMX treatment. Intracellular levels of the following proteins were assessed by flow cytometry in HSPCs: **(A)** MCL1; **(B)** BCL2; **(C)** BCL-XL.
Representative histograms are shown on the left, and relative expression changes (ratio of MFI between IR and non-IR conditions) are shown on the right.

**Supplemental Figure 16. Survivin expression in HSPCs.**

**(A–B)** CB CD34⁺ cells were cultured under the indicated conditions for up to 24 hours and subsequently analysed for intracellular Survivin expression in HSPCs. **(C–D)** CB CD34⁺ cells were cultured under the indicated conditions for up to 24 hours and subsequently analysed for intracellular Survivin expression in HSPCs.

| **Antigen** | **Clone** | **Fluorophore** | **Dilution** | **Manufacturer** | **Catalog number** |
| --- | --- | --- | --- | --- | --- |
| CD34 | 581 | FITC | 1-100 | Biolegend | 343504 |
|  | 581 | PE | 1-100 | Beckman Coulter | A07776 |
|  | 8G12 | APC | 1-100 | BD Bioscience | 345804 |
| CD38 | HB7 | PC7 | 1-100 | Biolegend | 356608 |
| CD45RA | HI100 | BV605 | 1-200 | Biolegend | 304134 |
| CD201 | RCR-401 | PE | 1-100 | Biolegend | 351904 |
|  | RCR-401 | APC | 1-100 | Biolegend | 351906 |
| CD33 | P67.6 | PE | 1-100 | Biolegend | 366608 |
| CD19 | SJ25C1 | APC | 1-100 | Biolegend | 363006 |
| Lineage cocktail (CD3/14/16/19/20/56) |  | FITC | 1-50' | Biolegend | 348801 |
| CD44 | IM7 | PE | 1-100 | Biolegend | 103023 |
| mSCA1 | D7 | FITC | 1-100 | Biolegend | 108105 |
| mCD140a | APA5 | PE | 1-100 | Biolegend | 135905 |
| mCD106 | 429 | APC | 1-100 | Biolegend | 105717 |
| mCD11b | M1/70 | FITC | 1-100 | Biolegend | 101205 |
| mCD31 | 390 | PC7 | 1-100 | Biolegend | 102417 |
| mCD34 | HM34 | APC | 1-100 | Biolegend | 128611 |
| mCD45 | 30-F11 | BV605 | 1-100 | Biolegend | 103140 |
| CD3 | REA613 | FITC | 1-100 | Miltenyi Biotec | 130-114-138 |
| CD45 | J33 | PC7 | 1-100 | Beckman Coulter | IM3548 |
| IgG1 | MOPC-21 | AF488 | 1-400 | Cell Signaling Technology | 4878S |
| IgG1 | DA1E | AF488 | 1-400/1-800 | Cell Signaling Technology | 2975S |
| BCL2 | 124 | AF488 | 1-100 | Cell Signaling Technology | 59422S |
| BCL-XL | 54H6 | AF488 | 1-100 | Cell Signaling Technology | 2767S |
| MCL1 | D2W9E | AF488 | 1-100 | Cell Signaling Technology | 58326S |
| P-CREB (S133) | 87G3 | AF488 | 1-200 | Cell Signaling Technology | 9187S |
| Survivin | 71G4B7 | AF488 | 1-200 | Cell Signaling Technology | 2810S |
| Annexin V |  | AF488 | 1-200 | Invitrogen | A13201 |
|  |  | APC | 1-200 | Invitrogen | A35110 |
| Sytox Blue |  |  | 1-1000 | Invitrogen | S34857 |
| Zombie NiR |  |  | 1-1000 | Biolegend | 423105 |
| TMRE |  |  |  | Abcam | Ab113852 |
| MitoTracker Green FM |  |  |  | Cell Signaling Technology | 9074 |
| Stem Cell Factor (SCF) |  |  |  | Peprotech | 300-07-100UG |
| FLT3 ligand (FLT3) |  |  |  | Peprotech | 300-19-100UG |
| Thrombopoietin (TPO) |  |  |  | Peprotech | 300-18-50UG |
| Non-Targeting Control siRNA |  |  |  | Lonza | D-001810-10-05 |
| BCL2 siRNA |  |  |  | Lonza | L-003307-00-0005 |
| BCL2L1 siRNA |  |  |  | Lonza | L-003458-00-0005 |
| MCL1 siRNA |  |  |  | Lonza | L-004501-00-0005 |
| ELISA MAX™ Deluxe Set Human IFN-γ |  |  |  | Biolegend | 430115 |
| ELISA MAX™ Deluxe Set Human TNF-α |  |  |  | Biolegend | 430215 |

**List of primers**

|  | Forward | Reverse |
| --- | --- | --- |
| GAPDH | TTC GTC ATG GGT GTG AAC CA | CTG TGG TCA TGA GTC CTT CCA |
| AREG | TGA GAT GTC TTC AGG GAG TG | AGC CAG GTA TTT GTG GTT CG |
| DUSP1 | TTC TTC CTC AAA GGA GGA TAC G | GTG GGG TAC TGC AGG AAC TG |
| FOS | CGT CTC CAG TGC CAA CTT CA | GGT CCG GAC TGG TCG AGA T |
| FOSB | TTG CAC CTT ACT TCC CCA AC | AGG AGT CCA CCG AAG ACA GA |
| VEGFA | CCA ATC GAG ACC CTG GTG | CAC ACA GGA TGG CTT GAA GA |
| CXCR4 | CCT ATG CAA GGC AGT CCA TGT | GGT AGC GGT CCA GAC TGA TGA |
| cJUN | TCG ACA TGG AGT CCC AGG A | GGC GAT TCT CTC CAG CTT CC |
| EREG | ATC ATG TAT CCC AGG AGA GTC CAG | GAA TCA CGG TCA AAG CCA CAT AT |
| INHBA | TCA CGT TTG CCG AGT CAG GAA C | TGA CAG GTC ACT GCC TTC CTT G |
| JOSD1 | TCC AGG ACA GCA ATG CCT TCA C | CAT GGT GTT TGG AGA CAA CCT CTG |
| PTGS2 | CCC TTG GGT GTC AAA GGT AA | GCC CTC GCT TAT GAT CTG TC |
| S1PR1 | ACG TAG GCT GTG GGA AGA TGA AG | TGG AAA CTT TGG CCT CAG CGA AG |
| ASPP1 | TTGTCCTCTCATTGCACG | AACTTACCCTCTCAGAGC |
| MDM2 | ATCAGCAGGAATCATCGGAC | CCAGGCTTTCATCAAAGGAA |
| PUMA | CCTGGAGGGTCCTGTACAATCT | GGACACAAGAAGAAAACCTTAATGC |
| NOXA | AGCTGGAAGTCGAGTGTGCT | TCCTGAGCAGAAGAGTTTGGA |
| CDKN1A | CGCGACTGTGATGCGCTAATG | GGAACCTCTCATTCAACCGCC |
| BCL2 | TGT GGA TGA CTG AGT ACC TGA ACC | GGAGAAATCCAGAGGCCGCAT |
| BCL-XL | GGA GAA CGG CGG CTG GGA TA | GGC CAC AGT CAT GCC CGT CA |
| MCL1 | CAT TCC TGA TGC CAC CTT CT | TCG TAA GGA CAA AAC GGG AC |

**Additional references**

1. Martin M. Cutadapt removes adapter sequences from high-throughput sequencing reads. EMBnet J. 2011;17(1):10.

2. Dobin A, Davis CA, Schlesinger F, Drenkow J, Zaleski C, Jha S, et al. STAR: Ultrafast universal RNA-seq aligner. Bioinformatics. 2013;29(1):15–21.

3. Anders S, Pyl PT, Huber W. HTSeq-A Python framework to work with high-throughput sequencing data. Bioinformatics. 2015;31(2):166–9.

4. Love MI, Huber W, Anders S. Moderated estimation of fold change and dispersion for RNA-seq data with DESeq2. Genome Biol. 2014;15(12):1–21.

5. Köster J, Rahmann S. Snakemake-a scalable bioinformatics workflow engine. Bioinformatics. 2012;28(19):2520–2.

6. Heinz S, Benner C, Spann N, Bertolino E, Lin YC, Laslo P, et al. Simple Combinations of Lineage-Determining Transcription Factors Prime cis-Regulatory Elements Required for Macrophage and B Cell Identities. Mol Cell [Internet]. 2010 May 28 [cited 2025 Nov 25];38(4):576–89. Available from: https://www.sciencedirect.com/science/article/pii/S1097276510003667?via%3Dihub

7. Subramanian A, Tamayo P, Mootha VK, Mukherjee S, Ebert BL, Gillette MA, et al. Gene set enrichment analysis: A knowledge-based approach for interpreting genome-wide expression profiles. Proc Natl Acad Sci U S A. 2005;102(43):15545–50.
