# Supplementary figures and images for: Mesenchymal stromal cells modulate survival and regeneration of human hematopoietic stem cells via PGE2/cAMP signaling
Source: Cell Death Dis. 2026 Mar 3;17(1):307. doi: 10.1038/s41419-026-08502-w (PMC13039678; doi:10.1038/s41419-026-08502-w)

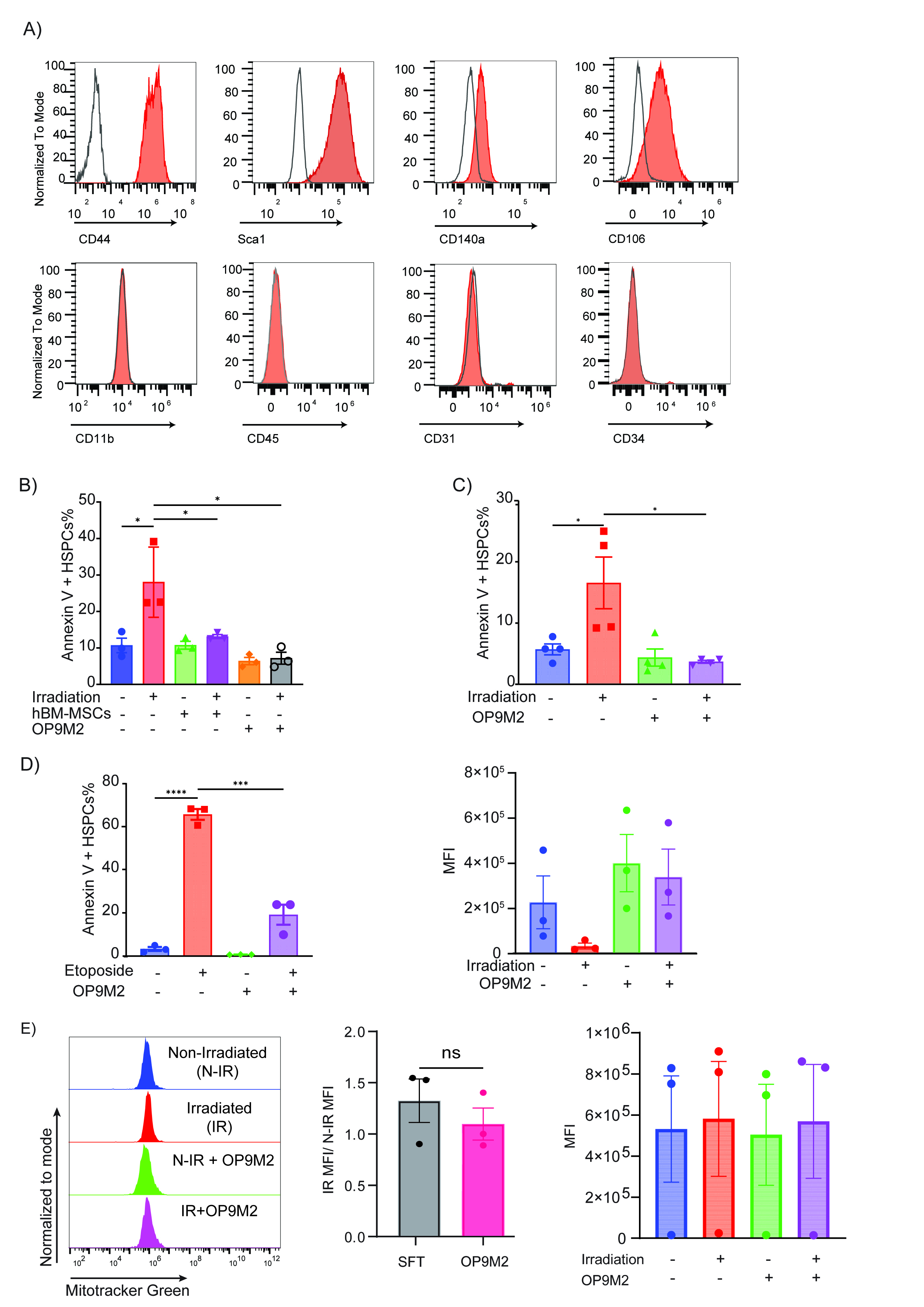

Supplement: Supplementary file 2 — Supplemental Figure 1 [file 41419_2026_8502_MOESM2_ESM.jpg]

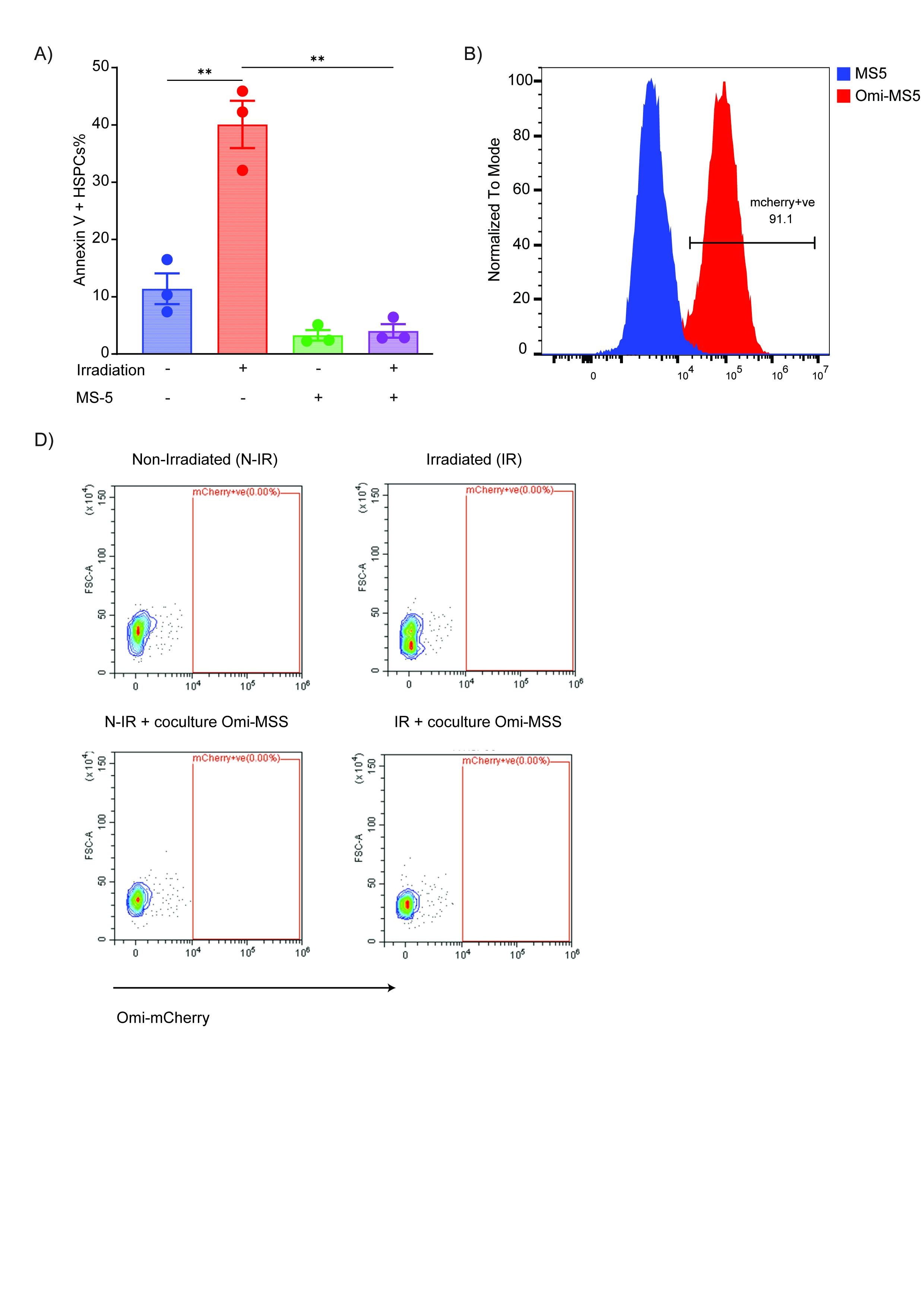

Supplement: Supplementary file 3 — Supplemental Figure 2 [file 41419_2026_8502_MOESM3_ESM.jpg]

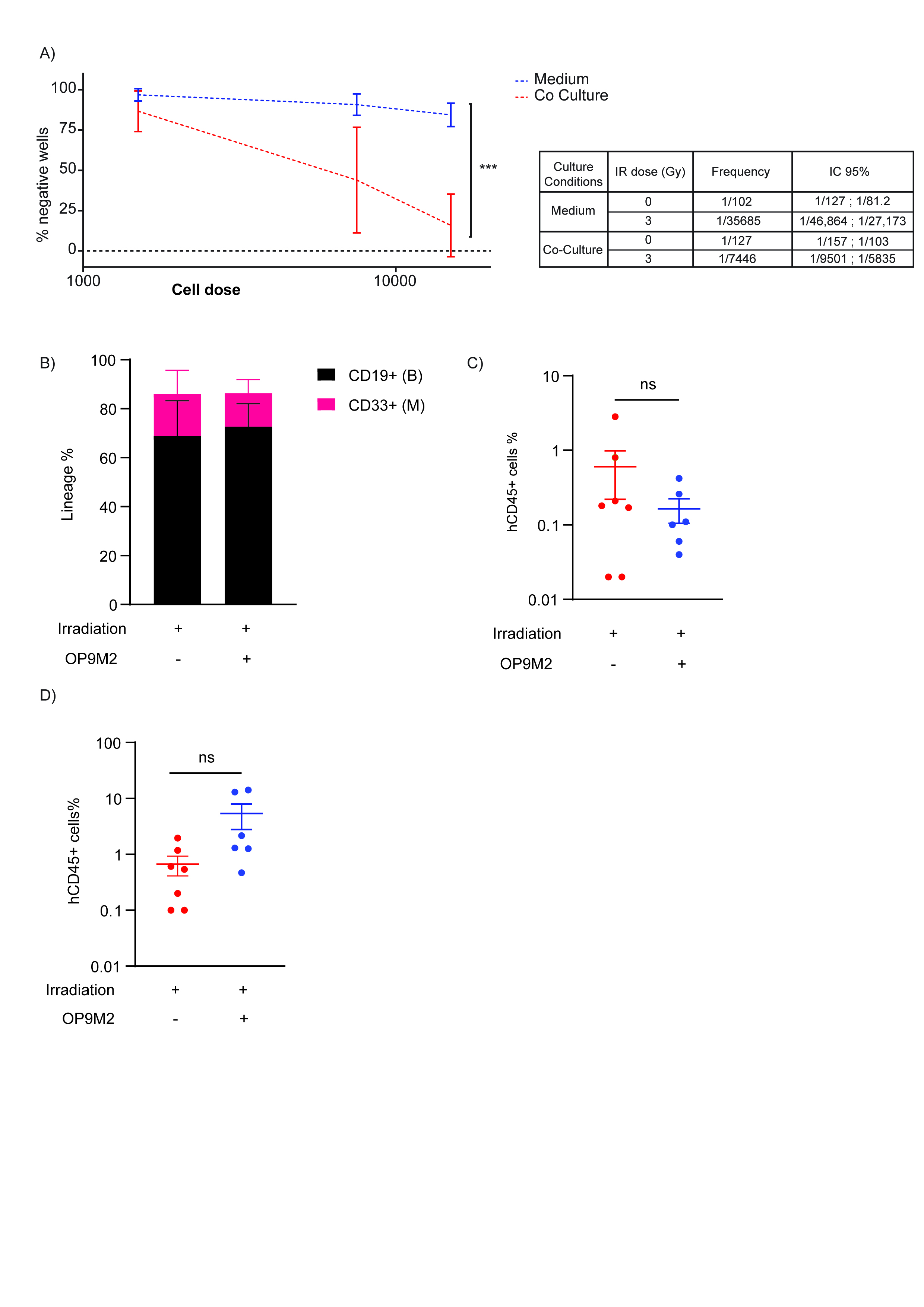

Supplement: Supplementary file 4 — Supplemental Figure 3 [file 41419_2026_8502_MOESM4_ESM.jpg]

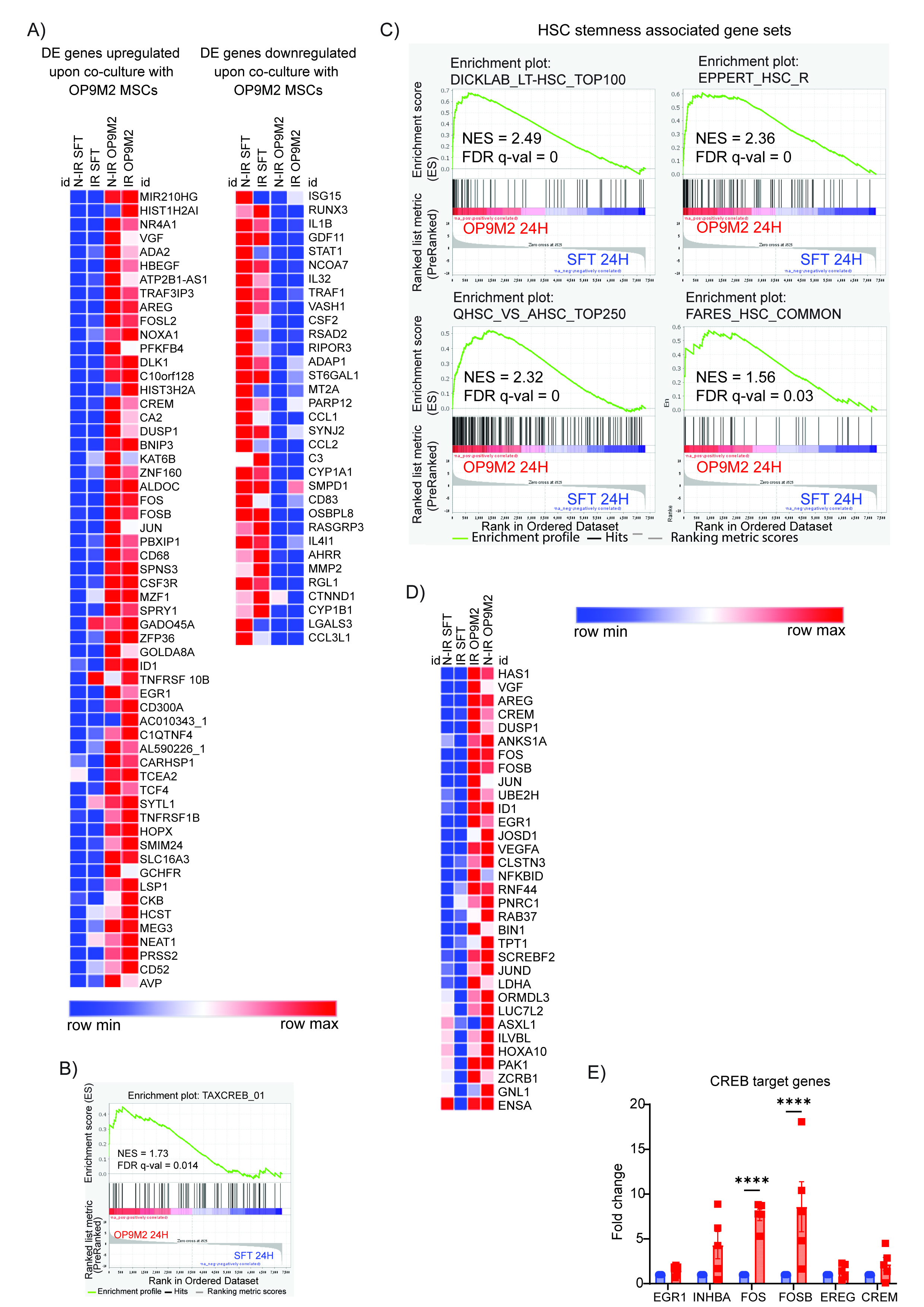

Supplement: Supplementary file 5 — Supplemental Figure 4 [file 41419_2026_8502_MOESM5_ESM.jpg]

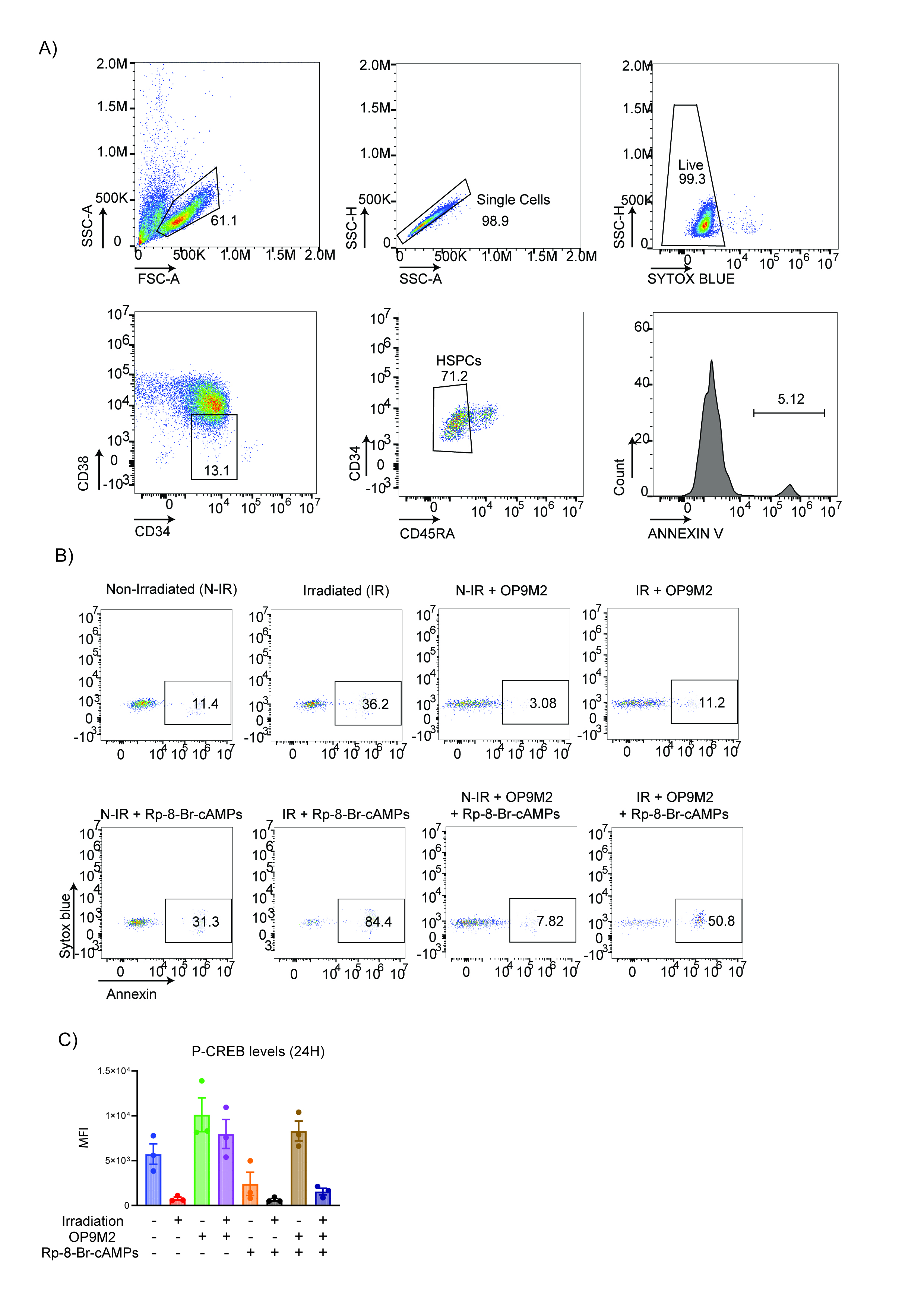

Supplement: Supplementary file 6 — Supplemental Figure 5 [file 41419_2026_8502_MOESM6_ESM.jpg]

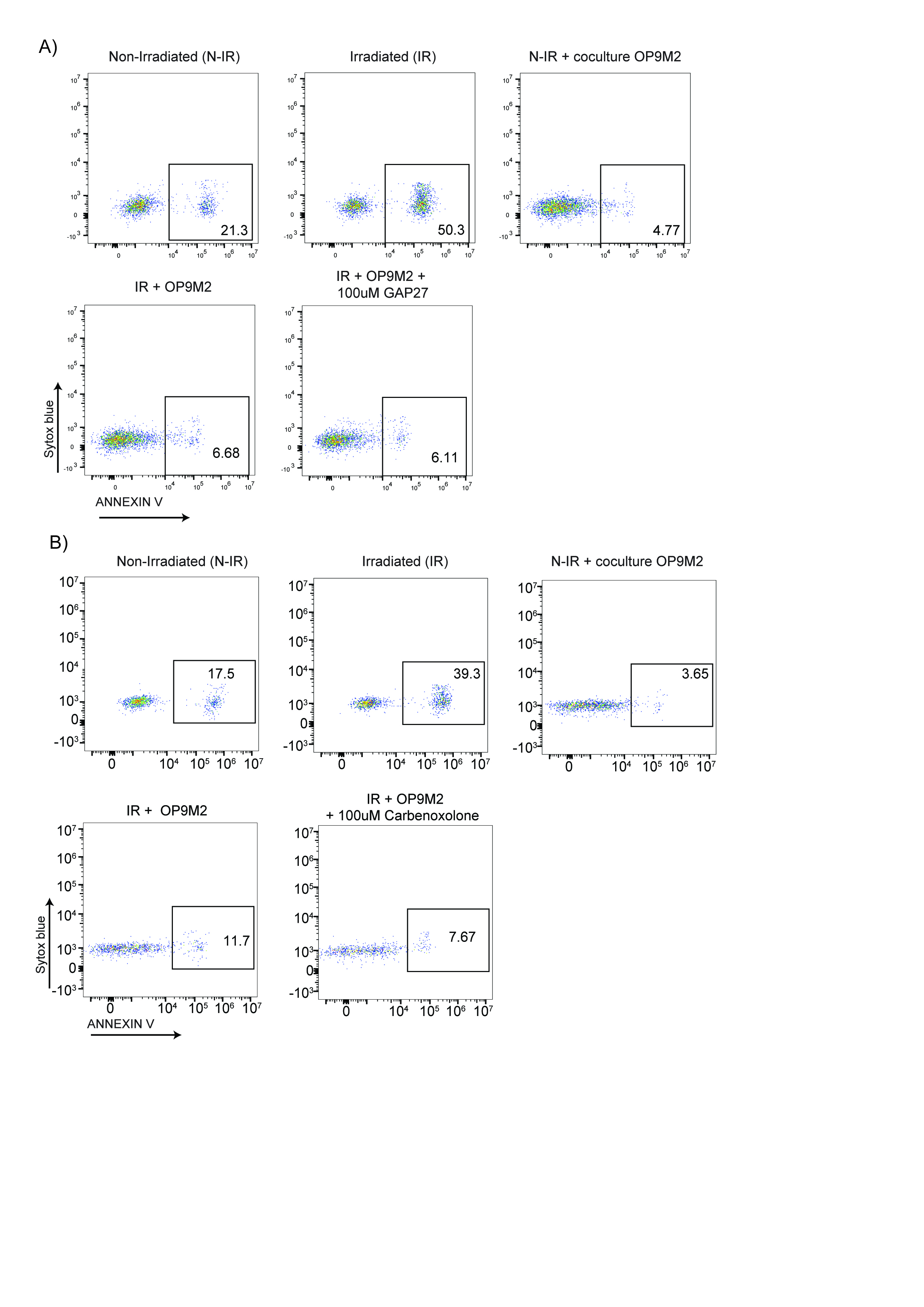

Supplement: Supplementary file 7 — Supplemental Figure 6 [file 41419_2026_8502_MOESM7_ESM.jpg]

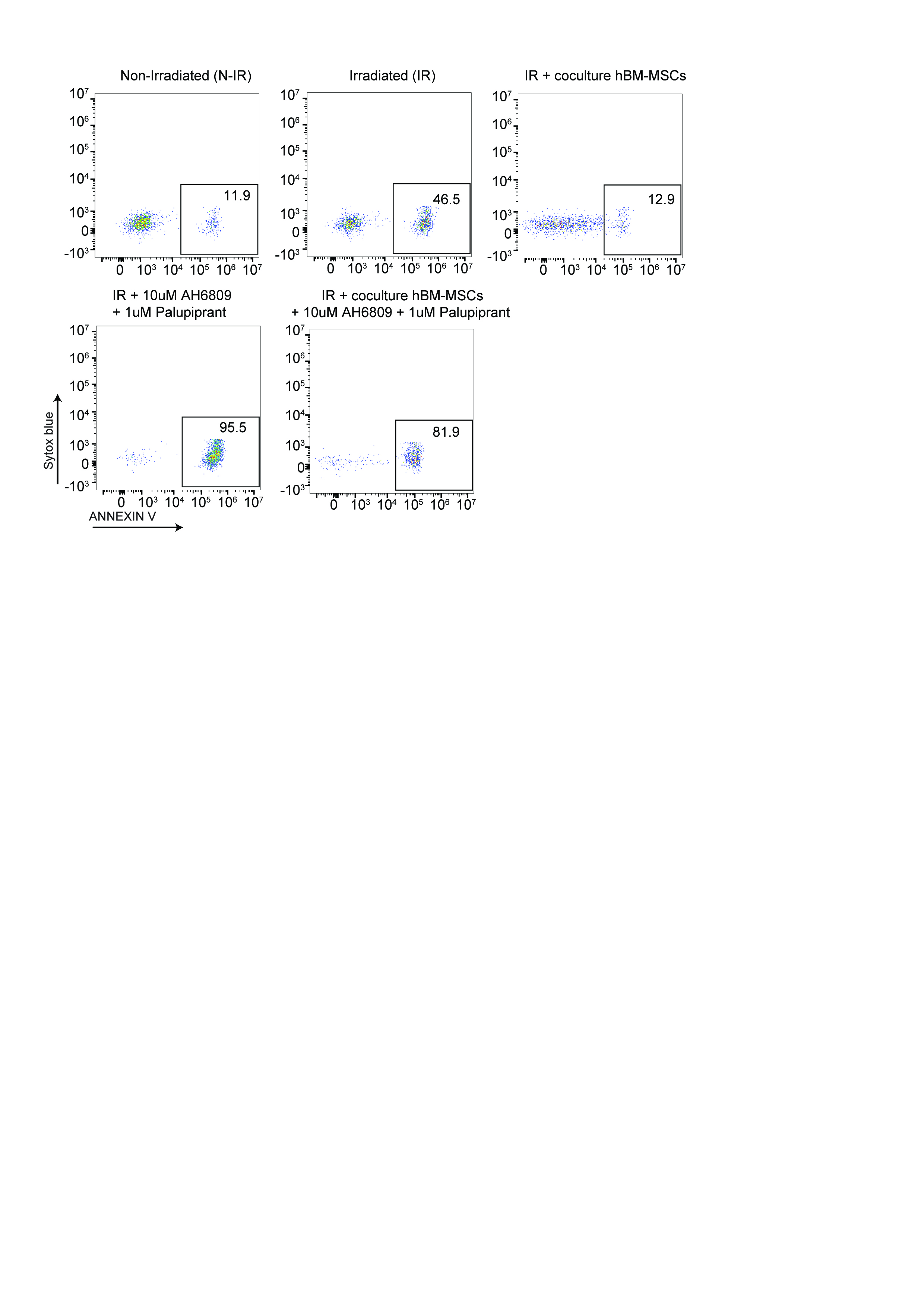

Supplement: Supplementary file 8 — Supplemental Figure 7 [file 41419_2026_8502_MOESM8_ESM.jpg]

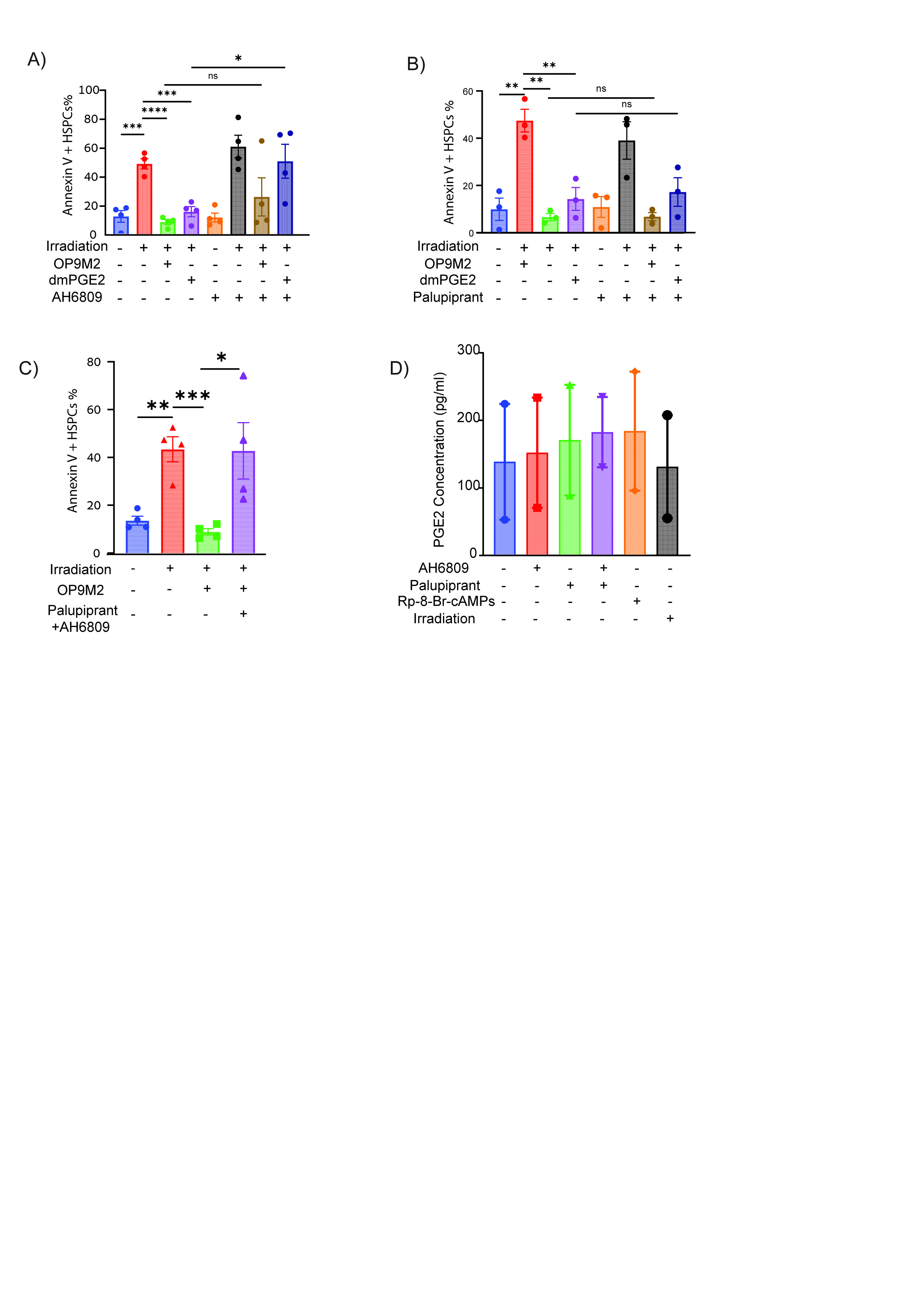

Supplement: Supplementary file 9 — Supplemental Figure 8 [file 41419_2026_8502_MOESM9_ESM.jpg]

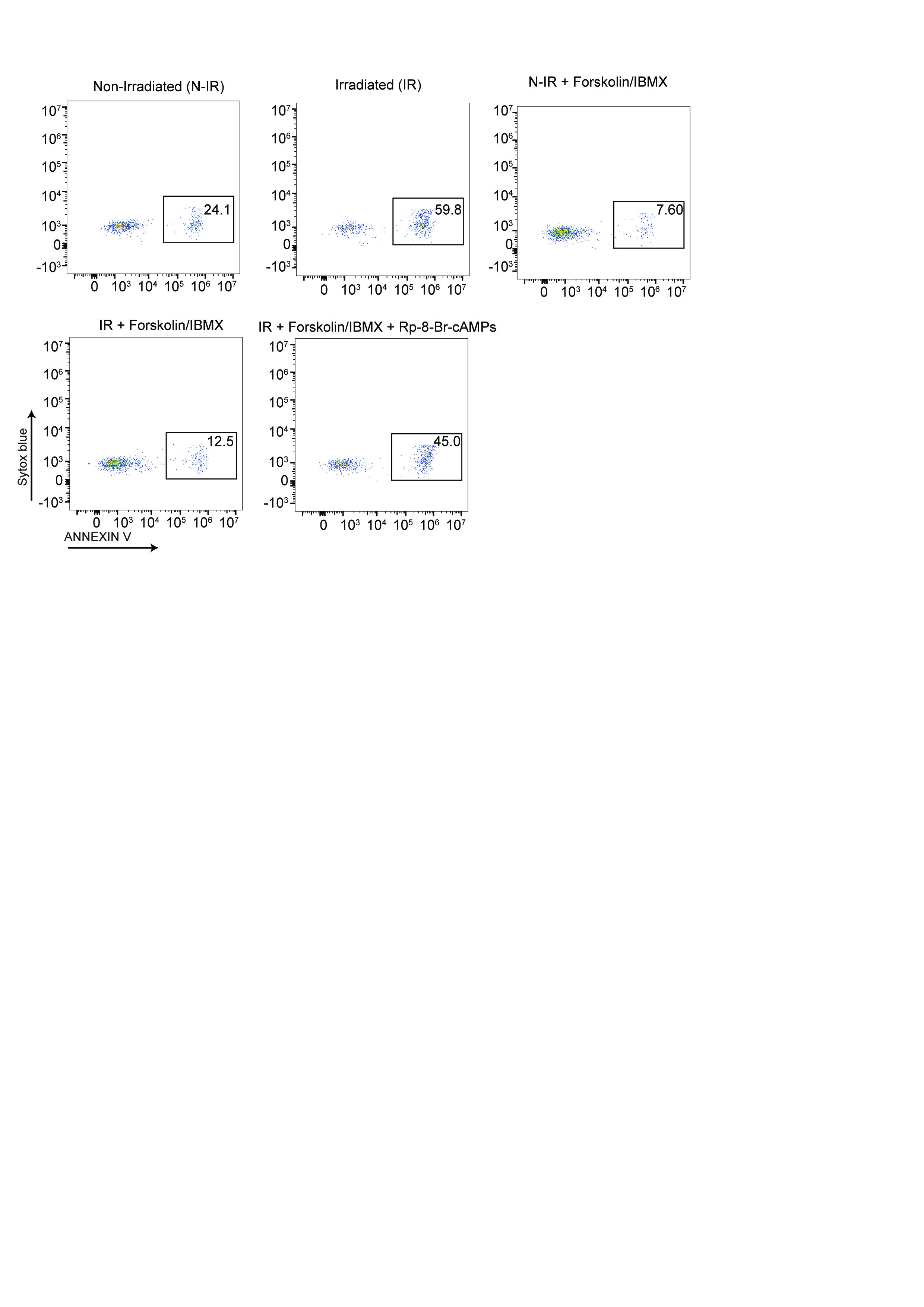

Supplement: Supplementary file 10 — Supplemental Figure 9 [file 41419_2026_8502_MOESM10_ESM.jpg]

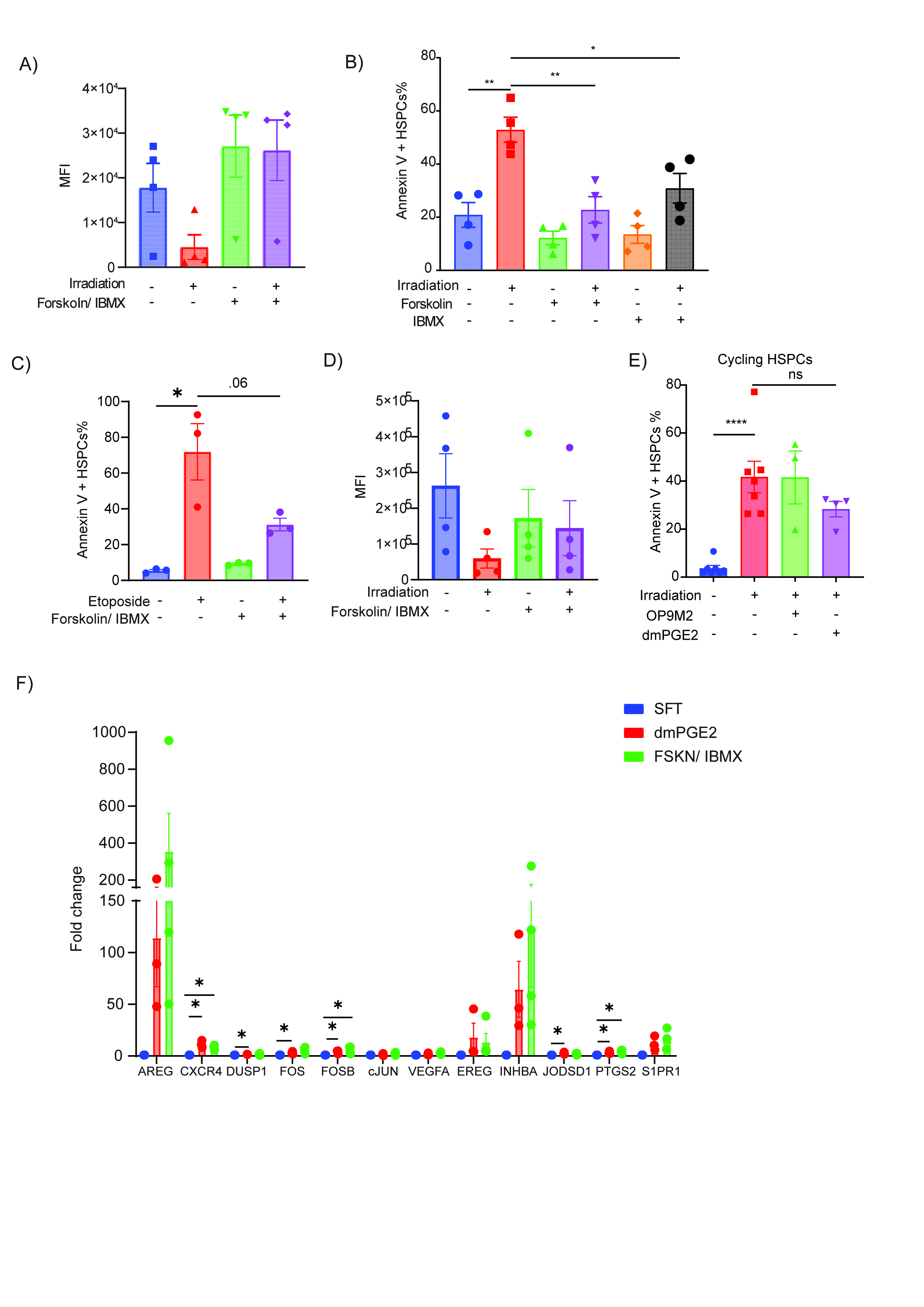

Supplement: Supplementary file 11 — Supplemental Figure 10 [file 41419_2026_8502_MOESM11_ESM.jpg]

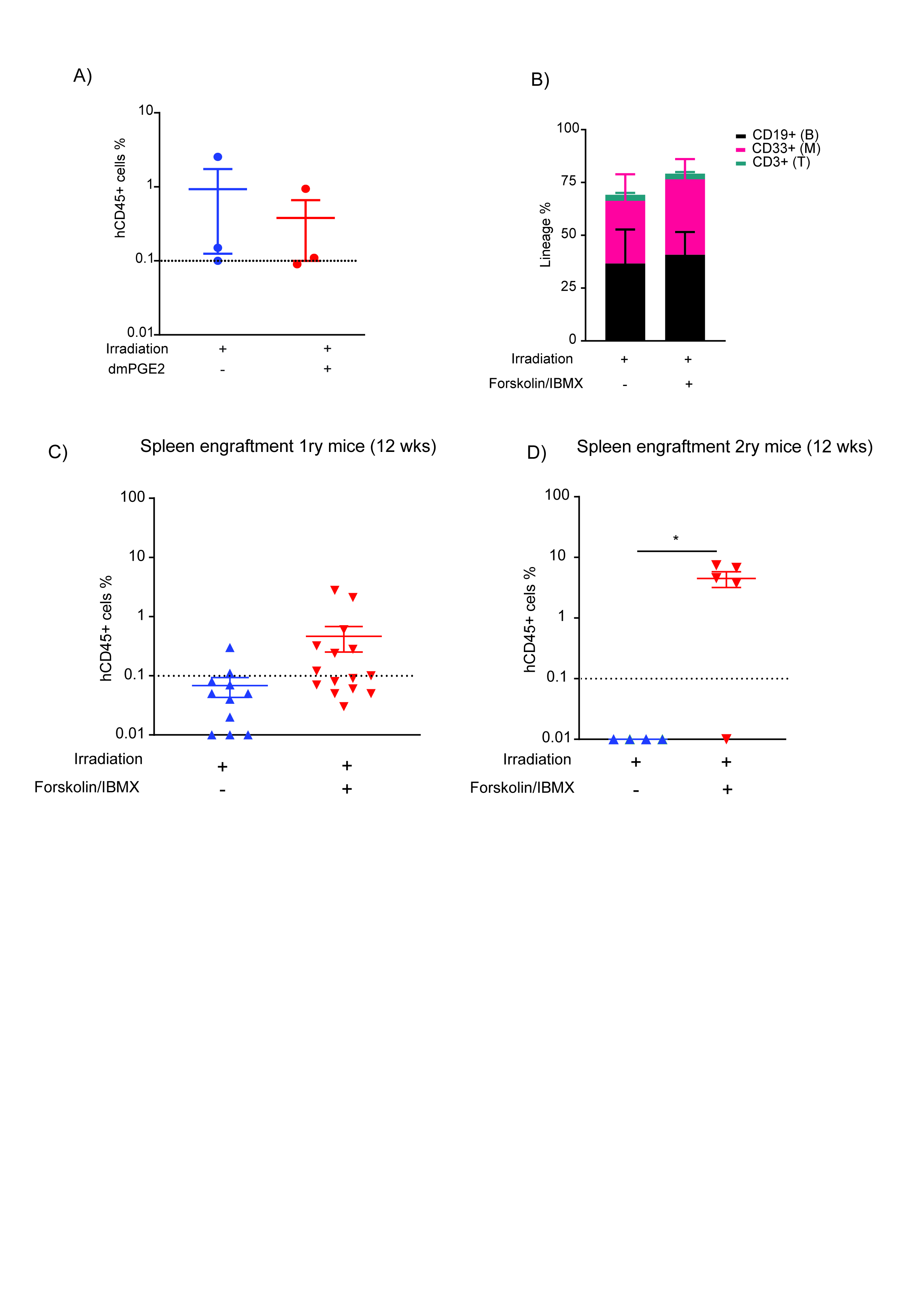

Supplement: Supplementary file 12 — Supplemental Figure 11 [file 41419_2026_8502_MOESM12_ESM.jpg]

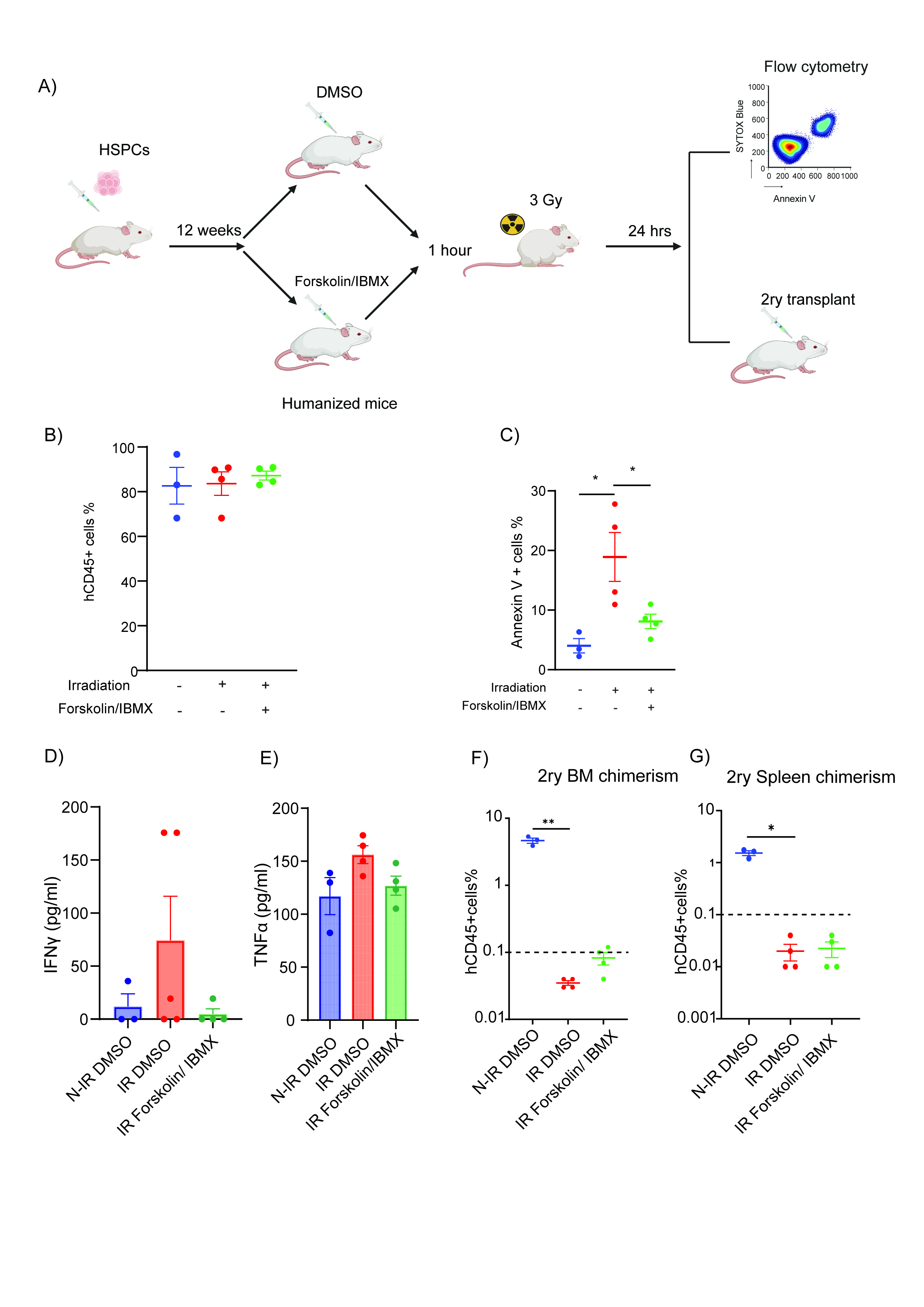

Supplement: Supplementary file 13 — Supplemental Figure 12 [file 41419_2026_8502_MOESM13_ESM.jpg]

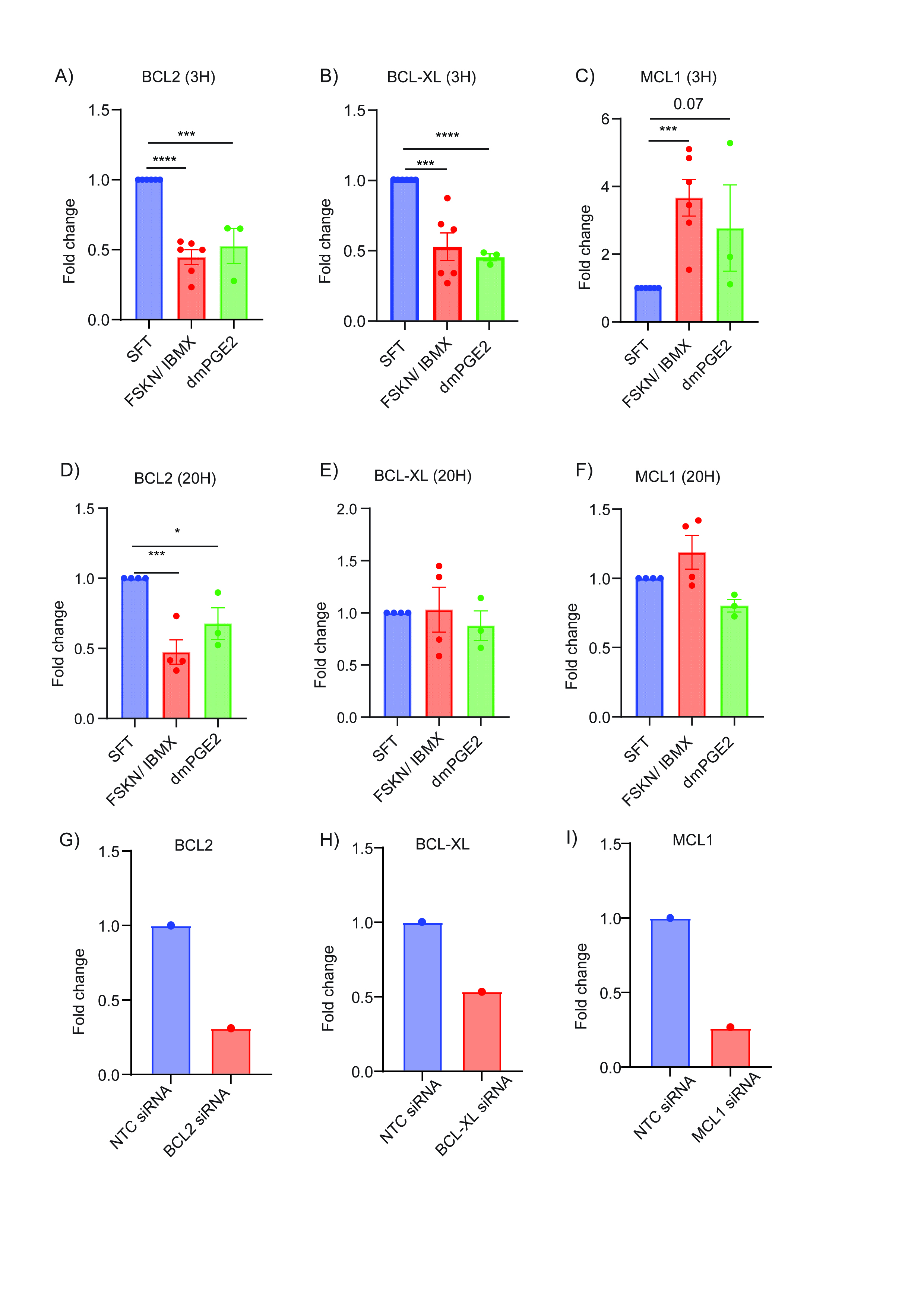

Supplement: Supplementary file 14 — Supplemental Figure 13 [file 41419_2026_8502_MOESM14_ESM.jpg]

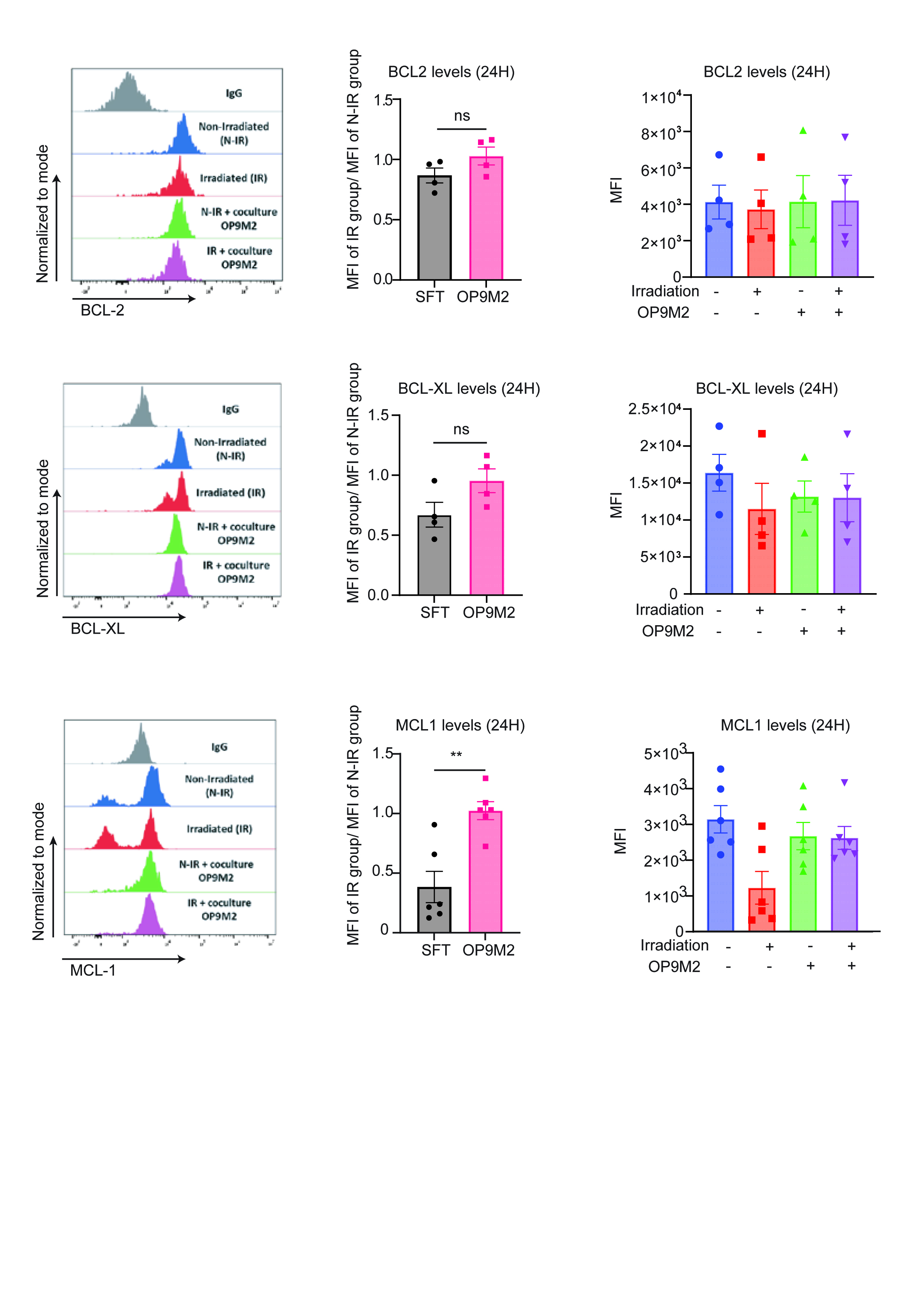

Supplement: Supplementary file 15 — Supplemental Figure 14 [file 41419_2026_8502_MOESM15_ESM.jpg]

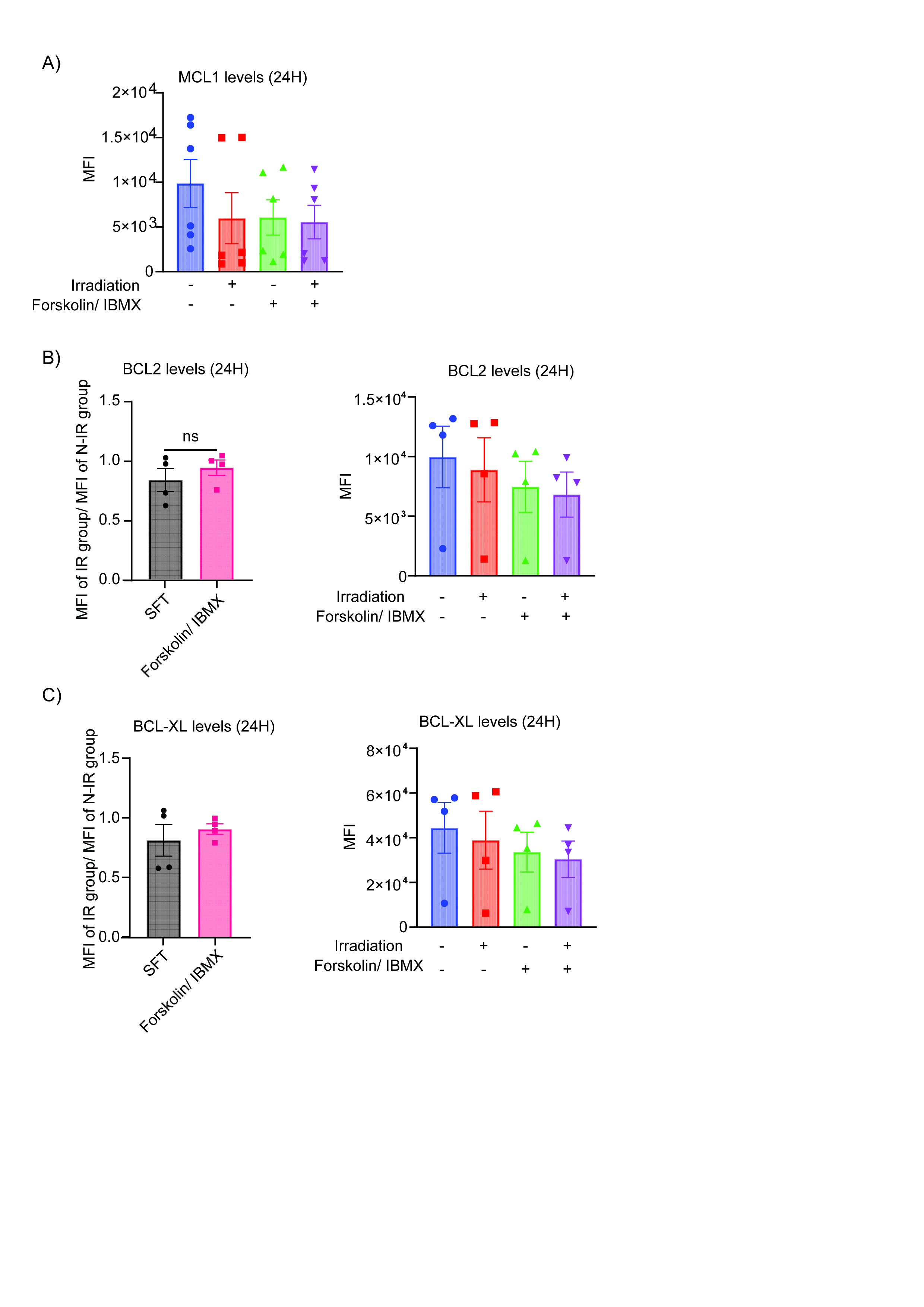

Supplement: Supplementary file 16 — Supplemental Figure 15 [file 41419_2026_8502_MOESM16_ESM.jpg]

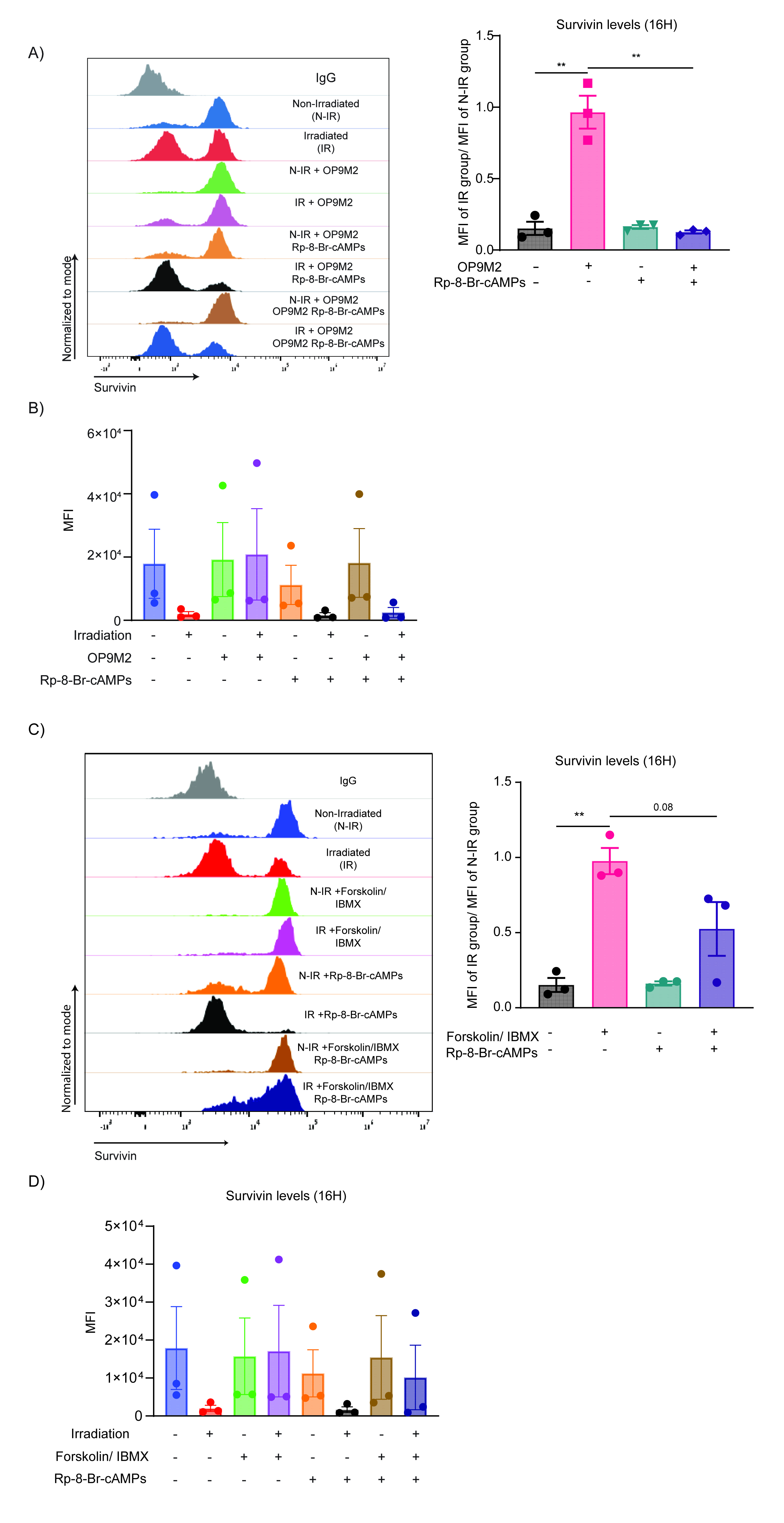

Supplement: Supplementary file 17 — Supplemental Figure 16 [file 41419_2026_8502_MOESM17_ESM.jpg]
